# Impaired nitrogenous waste clearance promotes hepatocellular carcinoma

**DOI:** 10.1126/sciadv.aec0766

**Published:** 2026-01-09

**Authors:** Xinlu Han, Jianliang Shen, Junrong Yan, Rahul Tacke, Weiwei Dai, Qingqing Mao, Heineken Queen Daguplo, Shuyang Liu, Ariful Islam, Tong Liu, Mark C. Koch, Richard Z. Lin, Hong Li, Tracy Anthony, Ping Xie, Lanjing Zhang, Shenglan Gao, M. Celeste Simon, Xin Chen, Jiekun Yang, Xiaoyang Su, Wei-Xing Zong

**Affiliations:** ^1^ Department of Chemical Biology, Ernest Mario School of Pharmacy, Rutgers University, Piscataway, NJ 08854, USA.; ^2^ Department of Genetics and Human Genetics Institute of New Jersey, Rutgers University, Piscataway, NJ 08854, USA.; ^3^ Department of Biosciences and Bioinformatics, Xi’an Jiaotong-Liverpool University, Suzhou, Jiangsu, China.; ^4^ Rutgers Cancer Institute, New Brunswick, NJ 08903, USA.; ^5^ Center for Advanced Metabolomics and Proteomics Research & Department of Microbiology, Biochemistry, and Molecular Genetics, Rutgers University–New Jersey Medical School, Newark, NJ 07103, USA.; ^6^ Department of Physiology and Biophysics, Stony Brook University, Stony Brook, NY 11794, USA.; ^7^ Northport VA Medical Center, Northport, NY 11768, USA.; ^8^ Department of Nutritional Science, Rutgers University, New Brunswick, NJ 08901, USA.; ^9^ Department of Cell Biology and Neuroscience, Rutgers University, Piscataway, NJ 08854, USA.; ^10^ Department of Pathology, Princeton Medical Center, Plainsboro, NJ, USA.; ^11^ Department of Cellular and Genetic Medicine, School of Basic Medical Sciences, Fudan University, Shanghai 200032, China.; ^12^ Abramson Family Cancer Research Institute, Department of Cell and Development Biology, University of Pennsylvania, Philadelphia, PA 19104, USA.; ^13^ Cancer Biology Program, University of Hawaii Cancer Center, Honolulu, HI 96813, USA.

## Abstract

In mammals, hepatic urea cycle enzymes (UCEs) convert ammonia, the toxic nitrogenous waste, into urea for excretion. In hepatocellular carcinoma (HCC), UCE expression is often heterogeneously repressed, but its role in tumorigenesis is unclear. We show that, as in patients, UCE expression is markedly reduced in multiple HCC mouse models, including those driven by oncogenic c-MET/β-catenin, leading to impaired ammonia clearance, altered amino acid metabolism, and increased pyrimidine synthesis. In contrast, UCE expression is largely preserved in c-MET/sgAxin1 tumors, allowing assessment of the consequences of UCE loss. Silencing individual UCEs increases ammonia burden and accelerates HCC with reprogrammed amino acid and pyrimidine metabolism, supporting a causal role for defective ammonia detoxification in oncogenesis. Notably, dietary protein restriction lowers hepatic ammonia and slows tumor growth. These findings establish a mechanistic link between nitrogen overload and hepatocarcinogenesis and highlight protein restriction as a feasible therapeutic strategy for patients with impaired nitrogenous waste handling.

## Teaser

Dietary protein restriction attenuates HCC growth.

## INTRODUCTION

Ammonia, the primary nitrogenous waste that is mainly produced by gut microbiome, is detoxified in liver via two major pathways: (i) conversion via the urea cycle (UC) into nontoxic urea for excretion; and (ii) assimilation into glutamate then glutamine by glutamate dehydrogenase (GDH) and glutamate ammonium ligase (GLUL, aka. glutamine synthetase, GS). UC enzymes (UCEs), including carbamoyl-phosphate synthetase 1 (CPS1), ornithine transcarbamylase (OTC), argininosuccinate synthetase (ASS), argininosuccinate lyase (ASL), and arginase (ARG), are expressed in the periportal area (zone 1) and midzonal area (zone 2) of the liver and help remove most ammonia from the portal blood. GLUL is expressed in the pericentral area (zone 3) and removes the remaining ammonia before the blood exists the central vein (hepatic vein). Genetic or pathological disruption of hepatic UCEs and GLUL leads to hyperammonemia, encephalopathy, and even death due to ammonia toxicity (*1*–*4*). Although increased blood ammonia level was noticed in patients with liver cancer in the early time (*5*), it has only recently become realized that hepatic ammonia level is associated with metabolic dysfunction–associated steatotic liver disease (MASLD) and hepatocellular carcinoma (HCC) (*6*, *7*). In preclinical mouse models, we and others showed that increased hepatic ammonia due to the loss of GLUL or increased ammonia import via SLC4A11 can promote HCC development (*7*, *8*).

In addition to the liver, other tissues also have at least partial UCE activities, which have been found involved in tumor growth/development in various organs, such as breast, pancreas, lung, and immune cells (*9*–*14*). Partially elevated UCE activities have been implicated in tumor promotion via mechanisms such as enhanced pyrimidine and polyamine production (*11*, *15*, *16*). In *Kras* or *LKB1*-mutated non–small cell lung carcinoma, up-regulation of CPS1 enhanced pyrimidine synthesis and promoted tumor growth (*11*). Trp^53^ was found to suppress the expression of UCEs including CPS1, OTC, and ARG1, which led to accumulation of ammonia that suppressed polyamine synthesis and cell proliferation (*16*).

In HCC, expression of UCEs is generally down-regulated, correlating with altered levels of UC metabolites including ornithine, arginine, and citrulline in the plasma of patients with HCC, and with worse prognosis (*15*, *17*–*22*). However, despite the strong correlation of UC deficiency in patients with HCC, the roles of the UC in its entirety and the individual UCEs remain largely elusive and controversial. For example, while CPS1 promotes tumorigenesis by maintaining the pyrimidine pool in certain cancer types (*11*), it has been shown that CPS1 deficiency can lead to increased fatty acid oxidation (*23*) and decreased Asp level hence elevated diacylglycerol (DAG)–protein kinase C (PKC) pathway (*24*). Moreover, ASS1 was reported to suppress HCC cell spheroid growth by activating the PERK/ATF4/CHOP endoplasmic reticulum stress pathway (*25*), and its down-regulation is associated with cisplatin-resistance in HCC (*26*). Furthermore, ASL has been mostly described to be protumorigenic in several types of cancer cells including liver cancer (*27*–*30*). Last, ARG1 was reported to promote HCC growth and metastasis by enhancing tumor cell epithelial-mesenchymal transition (*31*), whereas ectopic expression of ARG1 in a Tsc1/Pten DKO model suppressed HCC development by consuming arginine (*32*), and limiting arginine led to tumor cell growth arrest and apoptosis (*22*).

While these studies have started uncovering the impact of UC deficiency on HCC, many of them used cell culture, three-dimensional (3D) spheroids, and tumor cell transplantation systems, which do not faithfully recapitulate the unique metabolic and immunological features of the liver. Also, because the expression of UCEs is already suppressed in many HCC mouse models, it is empirically challenging to develop robust mouse models to conduct genetic silencing of the UCEs, and many studies relied on ectopic expression of the individual UCEs. In the current work, we compared the expression of the UCEs in several commonly used HCC mouse models and found that while the UCEs were severely suppressed by β-catenin, their expression remained relatively intact in the c-MET/sgAxin1 model. Using these models, we examined the impact of UCE down-regulation on HCC development. We then further tested the effect of low dietary protein intake on nitrogenous waste burden and HCC development.

## RESULTS

### Down-regulation of the UCEs is a widespread yet diversified event in patients with HCC and in mouse models

**Fig. 1.**
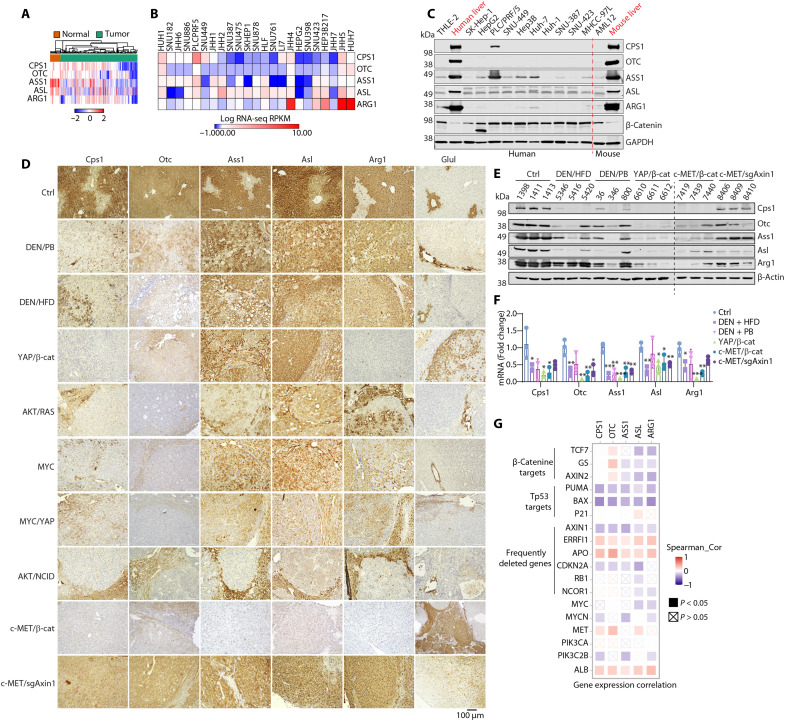
Differential expressions of UCEs in patients with HCC and mouse models. (**A**) Heatmap showing relative mRNA levels of the UCEs in normal liver and HCC tumor tissues using data obtained from TCGA database. (**B**) Heatmap showing mRNA level of UCEs in HCC cell lines with data obtained from CCLE database. (**C**) Western blot for UCEs in different HCC cell lines and human and mouse liver tissues. (**D**) IHC for UCEs in HCC mouse models driven by different carcinogens and oncogenes. For oncogene-driven liver cancer models, the mice were injected with constructs expressing oncogenes or sgRNA of tumor suppressor genes via SB-HTVI. (**E**) Western blot and (**F**) RT-qPCR for UCEs in several liver cancer mouse models. Data are shown as the means value ± SD in (F). *P < 0.05; **P < 0.01; ***P < 0.001; ns, not significant. Statistical significance was determined by unpaired Student’s *t* test. (**G**) Heatmap indicating the mRNA expression correlation between the UCEs and different oncogenic pathways, correlation was calculated using TIMER2.0.

Expression of the UCEs is generally down-regulated in HCC correlating with worse prognosis (fig. S1) (*15*, *17*–*22*). While the five UCEs were generally down-regulated in HCC, their expression patterns differed among patients (Fig. 1A). Similarly, analysis of the CCLE dataset showed that UCEs were generally down-regulated yet with a large variation in their levels in liver cancer cell lines (Fig. 1B), which was confirmed by immunoblotting of both human and mouse liver cell lines (Fig. 1C). Therefore, there exists a general yet diversified down-regulation of the UCEs in liver cancers. It remains to be determined whether and how altered expression of UCEs may affect HCC development.

Noting that the UCEs are down-regulated in human HCC yet with diverse expression patterns, we speculated that this phenomenon may be attributed, at least in part, by the highly heterologous oncogenic backgrounds in liver cancer. To this end, we examined the expression patterns of the UCEs in various mouse liver cancer models. These include the models induced by the carcinogen diethylnitrosamine (DEN) plus phenobarbital (PB) or high-fat diet (HFD), and by oncogenes or single-guide RNA (sgRNA) of tumor suppressors via the hydrodynamic tail vein injection with the Sleeping Beauty transposase (HTVI-SB) system. Immunohistochemistry (IHC) staining showed that while the UCEs CPS1, ASS1, ASL, and ARG1 were highly expressed in the periportal (zone 1) and midzonal areas (zone 2) in healthy control mice, they were expressed at various levels in the end-stage tumor tissues (Fig. 1D). This phenomenon was also observed by immunoblotting and quantitative reverse transcription polymerase chain reaction (qRT-PCR) in several models that we had freshly frozen liver tissues (Fig. 1, E and F). The widespread yet diverse down-regulation of UCEs mirrors the observation in patients with HCC (Fig. 1A), which was also consistent with TCGA analysis that showed various correlations between the UCEs and oncogenic backgrounds (Fig. 1G). In particular, while β-catenin showed a general inverse correlation with UCE expression, tumor suppressors Trp^53^, AXIN1, and CDKN2A also had inverse correlation with the UCEs, implying that their loss may lead to increased UCE expression (Fig. 1G).

Among the carcinogenic and oncogenic models, the c-MET/∆N90–β-catenin mice showed the most drastic suppression of UCEs, accompanied by elevated expression of Glul or GS (Fig. 1D). The c-MET/sgAxin1 mice showed only modestly down-regulated UCE expression and did not induce Glul expression as in the c-MET/β-catenin model (Fig. 1D). Axin1 is known to function as a tumor suppressor by scaffolding the β-catenin destruction complex to promote β-catenin degradation; therefore, sgAxin1 is expected to stabilize β-catenin and activate β-catenin signaling. The result that c-MET/sgAxin1 did not induce Glul and suppress UCEs is seemingly paradoxical but is consistent with previous reports that c-MET/sgAxin1 induces HCC independently of β-catenin (*33*) and does not induce GS expression (*34*). Bulk RNA sequencing (RNA-seq) comparing the c-MET/β-catenin and c-MET/sgAxin1 liver tissues showed that β-catenin and sgAxin1 had overlapping yet distinct expression profiles, with the canonical Wnt pathways more robustly enriched in the c-MET/β-catenin livers (fig. S2A). Close examination of the Wnt signaling pathway and ammonia-handling pathways also demonstrated common and distinct expression profiles between β-catenin and sgAxin1 (fig. S2, B and C). Therefore, the expression of UCEs is drastically suppressed in the c-MET/β-catenin model but only modestly altered in the c-MET/sgAxin1 model. This is consistent with the negative correlation of the UCEs and the canonical β-catenin signaling indicated by its transcription targets TCF7, GS, and AXIN2, which does not exist in the patients with AXIN1 loss of function (Fig. 1G). Because of the drastic difference between the c-MET/β-catenin and c-MET/sgAxin1 models in their regulation in UCE expression, in the following studies, we used the c-MET/β-catenin model to characterize the effect of UCE down-regulation and used the c-MET/sgAXIN1 model to study the effect of UCE silencing on nitrogen metabolism and HCC development.

### β-Catenin suppresses the expression of UCEs and increases ammonia burden

**Fig. 2.**
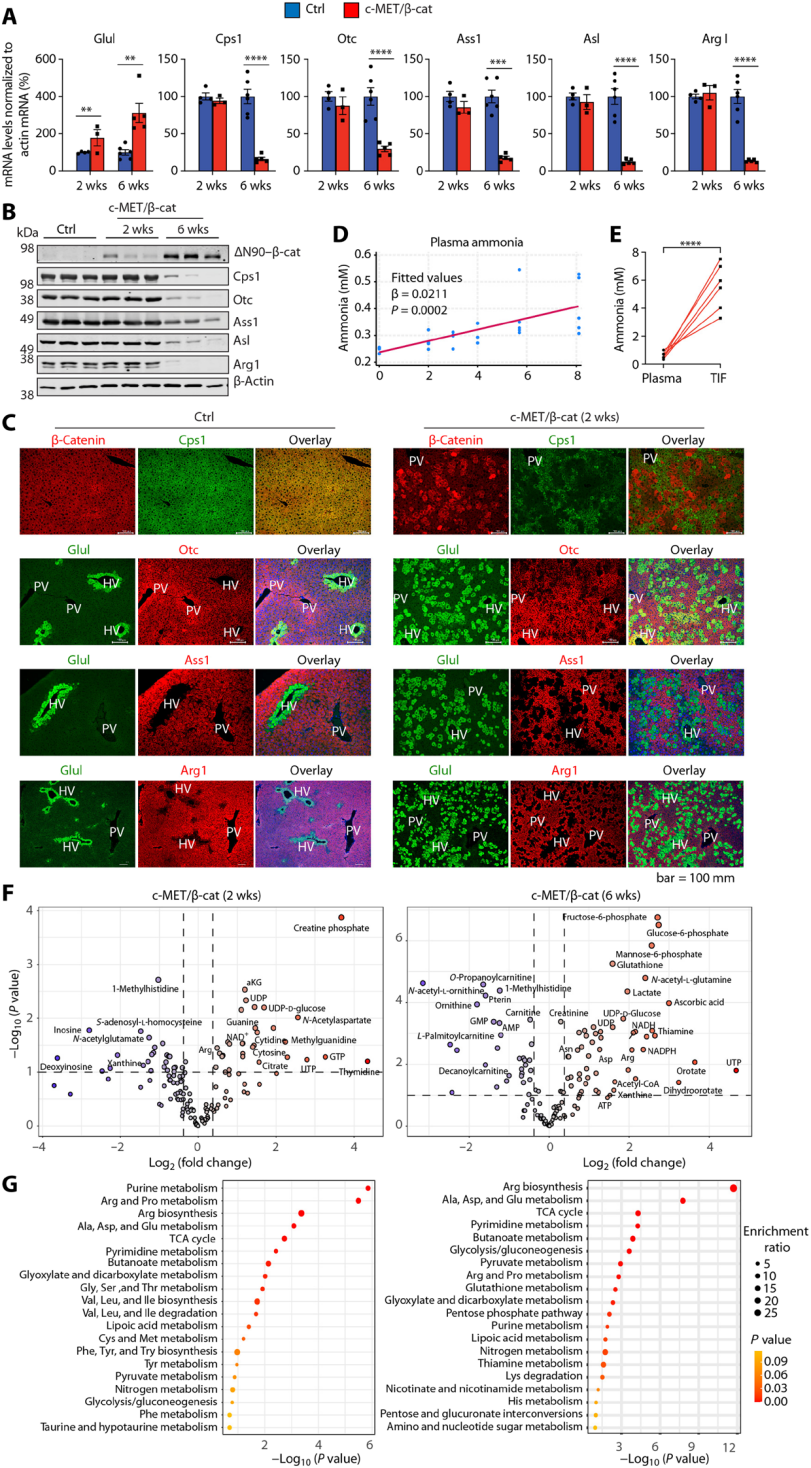
c-MET/β-catenin suppress expression of UCEs and cause increased ammonia burden. c-MET and ΔN90–β-catenin together with the Sleeping Beauty transposase plasmids were introduced into mice via HTVI-SB to induce HCC. (**A**) RT-qPCR and (**B**) Western blot for the UCEs in livers from the control (Vec) and c-MET/ΔN90–β-catenin mice 2 or 6 weeks (wks) after oncogene injection via SB-HTVI (*n* = 3 to 5 in each group). (**C**) Immunofluorescence costaining for β-catenin or Glul with the UCEs in liver from vector control and the c-MET/ΔN90–β-catenin mice 2 weeks after oncogene injection. PV, portal vein; HV, hepatic vein. Note the down-regulation of UCEs occurs in the zone 2 hepatocytes where ectopic β-catenin was expressed as indicated by β-catenin itself or the induction of Glul. (**D**) Orthogonal regression curve showing progressively increased plasma ammonia concentrations after c-MET/ΔN90–β-catenin injection for indicated times (*n* = 3 to 5 for each time point). (**E**) Ammonia level in plasma and tumor tissue internal fluid (TIF) at end point of the c-MET/ΔN90–β-catenin mice (*n* = 6). (**F**) Volcano plots showing the metabolic changes at 2 or 6 weeks upon c-MET/ΔN90–β-catenin injection compared with matched vehicle controls. (**G**) Pathway enrichment analysis of top 20 significantly changed metabolic pathways in each corresponding volcano plot. Data were analyzed by MetaboAnalyst 6.0 using KEGG metabolite sets library. Data are shown as the means value ± SD. **P < 0.01; ***P < 0.001; ****P < 0.0001; ns, not significant. Statistical significance was determined by unpaired Student’s *t* test in (A) and paired Student’s *t* test in (E).

The down-regulation of UCEs in patients with HCC and in various mouse models might be a direct transcription event downstream of oncogenic activation or a consequence of stress response to liver injury during carcinogenesis. In healthy livers, hepatocyte partitioning of the UCEs is transcriptionally regulated by the Wnt/β-catenin pathway, which is suppressed in the periportal (zone 1) and midzonal (zone 2) areas by high levels of adenomatous polyposis coli expression and is activated in the pericentral zone (zone 3) (*35*, *36*). While the homeostatic level of Wnt/β-catenin is developmentally important, hyperactivation of β-catenin signaling via genetic mutations or other oncogenic pathways is a prevalent causative event in HCC. Because UCEs were drastically suppressed in the end-point tumors in the c-MET/β-catenin model (Fig. 1, D to F), we further examined this model more closely for UCE expression and ammonia clearance.

c-MET and the constitutively active N-terminal deletion mutant of β-catenin (∆N90–β-catenin) were introduced into mice via HTVI-SB to induce HCC, as previously described (*37*, *38*). This model develops HCC with a latency of 8 to 12 weeks in mice with 129/Ola and C57BL/6 mixed background that we use (*8*). We harvested the liver tissues at various time points after oncogene injection. RT-qPCR and immunoblotting showed that while the transcription target of β-catenin Glul was significantly higher at both early (2 weeks) and late (6 weeks) time points after oncogene injection (Fig. 2A), down-regulation of the UCEs was more obvious at the 6-week time point (Fig. 2, A and B). Closer examination of the 2-week liver tissues showed strong induction of Glul and suppression of the UCEs in the hepatocytes with oncogene expression (Fig. 2C), indicating the suppression of the UCEs is an early event of β-catenin activation. Consistent with the decreased UCE expression, the c-MET/β-catenin mice showed a progressive increase of ammonia level in the peripheral blood (Fig. 2D), which was further elevated in the liver tumor interstitial fluid (TIF) (Fig. 2E).

We then performed untargeted metabolomic analysis by liquid chromatography–mass spectrometry (LC-MS) to examine the metabolic changes induced by c-MET/β-catenin at the 2-week and 6-week time points comparing with the basal state mice (without oncogenes). The most significant changes were the increase in metabolites related to nucleotides, carbohydrates, and amino acids at 2 weeks, which became more drastic at 6 weeks including increased arginine, aspartate, pyrimidine precursors dihydroorotate and orotate, and decreased ornithine (Fig. 2F). Metabolic pathway enrichment analysis using KEGG metabolite sets library further indicated that c-MET/β-catenin expression led to metabolic reprogramming related to arginine and other amino acids, nucleosides, and carbohydrates (Fig. 2G). Bulk RNA-seq of the liver tissues at early time points after c-MET/β-catenin expression (2 and 3 weeks) showed changes of gene expression of cell growth/migration, carbohydrate metabolism, and lipid metabolism (fig. S3).

### β-Catenin leads to abnormal amino acid metabolism and pyrimidine synthesis

**Fig. 3.**
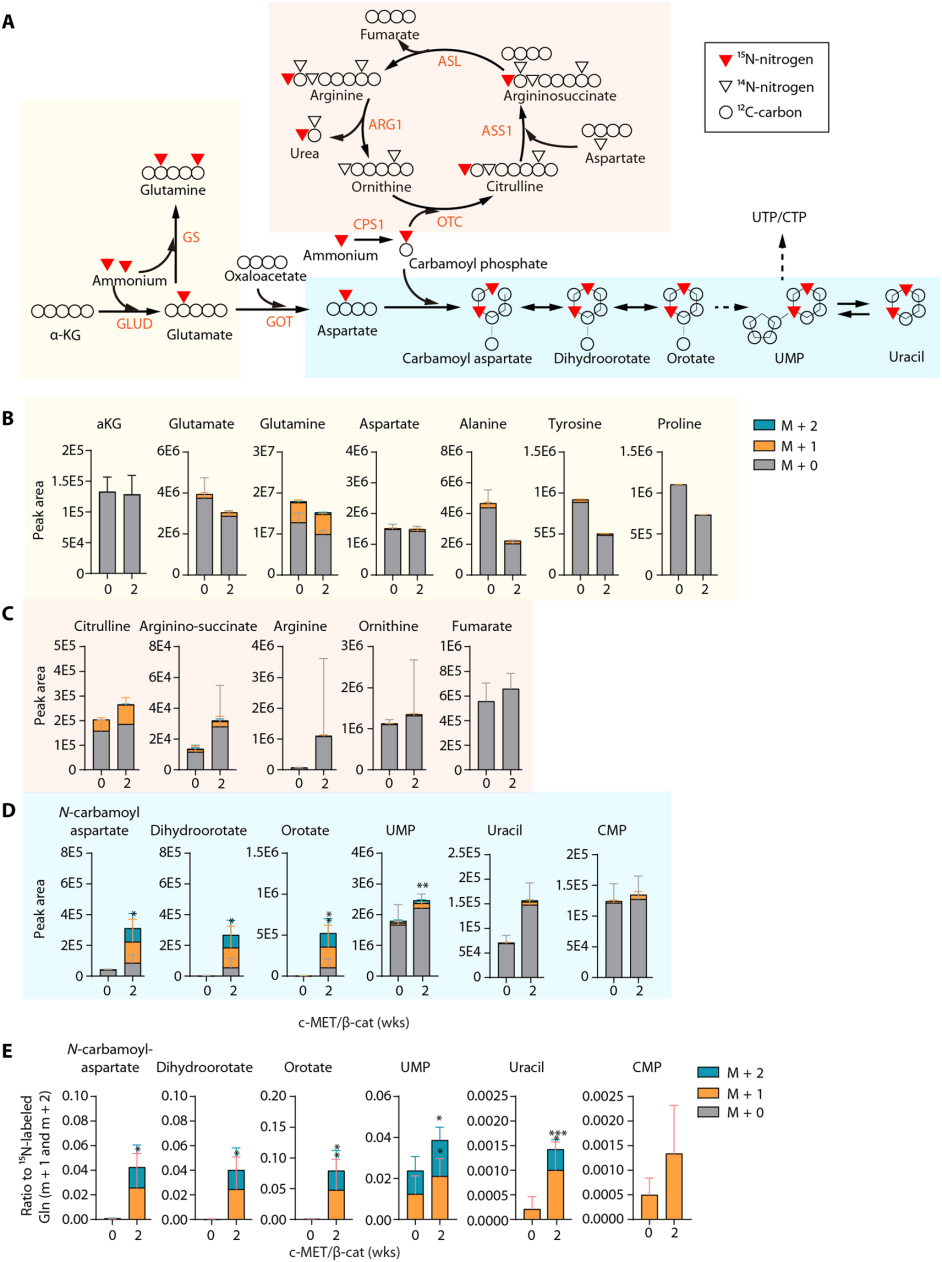
^15^N-NH_4_Cl tracing shows incorporation of ^15^N into pyrimidine synthesis. (**A**) Schematic diagram of the metabolic fates of ammonia in the liver. The three main pathways (glutamate/glutamine synthesis, UC, and pyrimidine synthesis) are highlighted with different background colors. (**B** to **D**) ^15^N-ammonium chloride was injected via intraperitoneal bolus to the vector or mice expressing c-MET/ΔN90–β-catenin for 2 weeks (*n* = 6 in each group). Liver tissues were harvested after 30 min and subjected to LC-MS. Abundance of ^15^N-labeled and total metabolites in the glutamate/glutamine route (B), UC route (C), and pyrimidine synthesis (D) are shown. (**E**) Ratio of indicated ^15^N-labeled isotopologs to ^15^N-labeled glutamine. Note that oncogene expression led to increased ^15^N-incorporation into the pyrimidine synthesis pathway in the oncogene mice. Data are shown as the mean value ± SD. *P < 0.05; **P < 0.01; ***P < 0.001; ns, not significant. Statistical significance was determined by unpaired Student’s *t* test.

Since ammonia clearance was defective in c-MET/β-catenin–transfected livers (Fig. 2, D and E), we went on the test whether the increased ammonia burden may contribute to oncogenesis. To further delineate the metabolic changes upon β-catenin activation at early stages, we performed stable isotope tracing using ^15^N-labeled ammonium (^15^N-NH_4_Cl for intraperitoneal bolus) in mice injected with c-MET/β-catenin to examine the three metabolic fates of hepatic ammonia: (i) assimilation into glutamate and glutamine; (ii) entry to the UC via carbamoyl phosphate; and (iii) entry to pyrimidine synthesis via the carbamoyl phosphate-orotate pathway (Fig. 3A). Considering that some of the changes at late-stage tumor development may not be the causal effect (6-week time point in Fig. 2F), we used mice at the early 2-week time point.

For the glutamate/glutamine ammonia assimilation route, enrichment of ^15^N-glutamate was about 5%, with slight yet nonsignificant decrease upon oncogene expression for 2 weeks (Fig. 3B). Consistently, glutamate-derived nonessential amino acids (NEAAs) Asp, Ala, Tyr, and Pro showed either slight decrease or non-significant changes in ^15^N labeling (Fig. 3B). The enrichment of ^15^N-glutamine was about 30% with approximately 98% labeled fraction being m + 1 and 1.6% being m + 2 at in both control and oncogene-expressing mice (Fig. 3B). The near 30% labeling of glutamine is consistent with the literature and our previous work and can be used as an indication of equal ^15^N labeling efficiency (*8*, *39*). Together, these data indicate that GLUL (GS)–mediated glutamine production is a more dominant way of ammonia assimilation than glutamate production mediated by GDH and that the glutamate/glutamine ammonia assimilation was not significantly altered by c-MET/β-catenin, at least at the early stage of hepatocyte transformation.

For the UC pathway, there were various trends of increase of the pool size and ^15^N-labeling of UC metabolites citrulline, argininosuccinate, fumarate, arginine, and ornithine (Fig. 3C). These changes were consistent with defective UC, although the relatively minor changes could be due to only a small portion of hepatocytes having received oncogenes.

Strikingly, a marked increase of the de novo pyrimidine synthesis pathway was observed 2 weeks after oncogene expression. Increased labeled and pool sizes of *N*-carbamoyl-aspartate, dihydroorotate, orotate, uracil, and cytidine 5′-monophosphate (CMP) were observed (Fig. 3D). Using ^15^N-labeled glutamine as an indicator of labeling efficiency, the ratio of labeled pyrimidine metabolites to glutamine also indicates a strong up-regulation of pyrimidine synthesis (Fig. 3E), consistent with the bulk metabolomics data (Fig. 2F). Therefore, c-MET/β-catenin activation suppresses the expression of the UCEs and leads to increased ammonia burden that contributes to abnormal amino acid and pyrimidine metabolism.

### Silencing UCEs in the c-MET/sgAxin1 model accelerated HCC development

We next sought to determine the effect of UCE deficiency on HCC development. Notably, most of the HCC mouse models that we have tested showed drastically decreased expression of UCEs (Fig. 1, D to F), making them inadequate for testing the effect of UCE loss of function. The c-MET/sgAxin1 model, which did not drastically down-regulate the expression of UCEs (Fig. 1, D to F, and fig. S2), was ideal for examining the effect of UCE silencing.

**Fig. 4.**
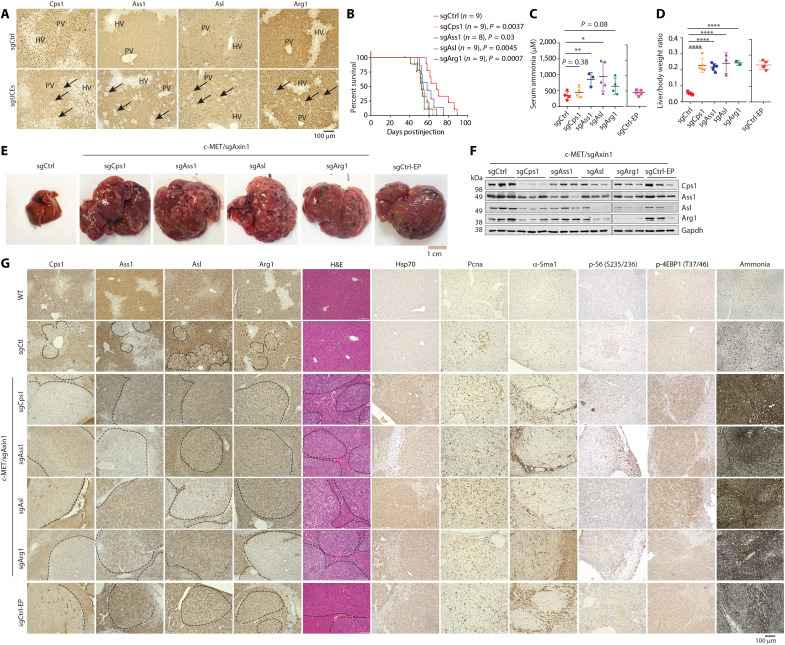
Silencing UCEs culminates HCC in the c-MET/sgAxin1 model. sgControl (sgCtrl) or sgUCE (Cps1, Ass1, Asl, and Arg1) were coinjected with c-MET/sgAxin1 via SB-HTVI into 8-week-old male mice. (**A**) IHC for the UCEs in sgCtrl or sgUCEs liver 2 weeks after plasmid injection. Arrow points to the cell with successful silencing of the respective UCE. PV, portal vein; HV, hepatic vein (central vein). (**B**) Kaplan-Meier survival curves of sgCtrl and sgUCEs mouse post–SB-HTVI. (**C**) sgUCE mice were harvested at their end points. Age-matched and end-point sgCtrl (sgCtrl0EP) mice were also harvested (*n* = 3 to 5 in each group). Plasma ammonia level was measured. (**D**) Liver/body weight ratio of sgCtrl and sgUCEs mice (*n* = 3 to 5 in each group). (**E**) Representative gross images of livers from the sgCtrl and sgUCE groups harvested at the end point of the sgUCE mice. End-point sgCtrl (sgCtrl-EP) mice were also shown. (**F**) Western blots for the UCEs in sgCtrl, sgUCE, and sgCtrl-EP liver. The sgCtrl and sgUCE mice were harvested at end points of the sgUCE mice. Note that all mice were coinjected with c-MET/sgAxin1. (**G**) IHC for the UCEs, Hsp70, Pcna, α-Sma, p-S6 (S235/236), and p-4EBP (T37/46); hematoxylin and eosin (H&E) staining and Nessler’s staining for ammonia in sgCtrl and sgUCEs livers. Tumor boundaries are delineated with dashed lines. Data are shown as the mean value ± SD [(C) and (D)]. *P < 0.05; **P < 0.01; ****P < 0.0001; ns, not significant. Statistical significance was determined by log-rank test in (B) and unpaired Student’s *t* test in (C) and (D).

sgRNA for each UCEs were cloned into the CRISPR-Cas9–containing pX330 plasmid and introduced into mouse livers via the HTVI-SB system. Four UCEs CPS1, ASS1, ASL, and ARG1 were successfully silenced in sporadic hepatocytes confirmed by IHC (Fig. 4A). We then injected the UCE sgRNAs together with c-MET/sgAxin1 to determine the effect of UCE silencing on HCC development. Strikingly, knocking out each of the four UCEs significantly shortened the life span of the mice (Fig. 4B), which was accompanied by increased blood ammonia levels (Fig. 4C). An increased tumor burden was observed in all four knockouts (Fig. 4, D and E). When the sgUCEs mice reached end points, their livers showed various numbers of nodules on the surface and enlargement (hepatomegaly) while the time-matched sgControl (c-MET/sgAxin1 only) showed normal looking liver. The hepatic nodules in the sgUCE mice were yellow tan in color, varied by size and had distorted the liver contour (Fig. 4E). Immunoblotting and IHC of the end-point liver tissues confirmed persistent silencing of the UCEs (Fig. 4, F and G).

Microscopically, the tumor tissues showed solid-type HCC that was poorly differentiated, characterized by marked nuclear atypia, thickened liver plates, lack of bile ducts, and steatotic changes at various levels (Fig. 4G, IHC). The sgUCE mice also showed high levels of an HCC marker HSP70 and faster proliferation of the hepatocytes than the mice with only c-MET/sgAxin1, indicated by the proliferation marker proliferating cell nuclear antigen (PCNA) and the mammalian target of rapamycin C1 (mTORC1) signals (phospho-S6 S235/236 and phospho-4EBP1 T37/46) (Fig. 4G). Tissue fibrotic response was also markedly increased in sgUCE mice as indicated by the expression of α–smooth muscle actin (α-SMA) (Fig. 4G). Consistent with the elevated ammonia level detected by the colorimetric assay (Fig. 4C), the sgUCE liver tissues displayed high ammonia levels detected by Nessler’s staining (Fig. 4G). These data indicate that deficiency of each of the UCEs CPS1, ASS1, ASL, or ARG1 all accelerated c-MET/sgAxin1–induced HCC development in mice.

### Silencing UCEs leads to reprogramming of amino acid metabolism and pyrimidine synthesis

**Fig. 5.**
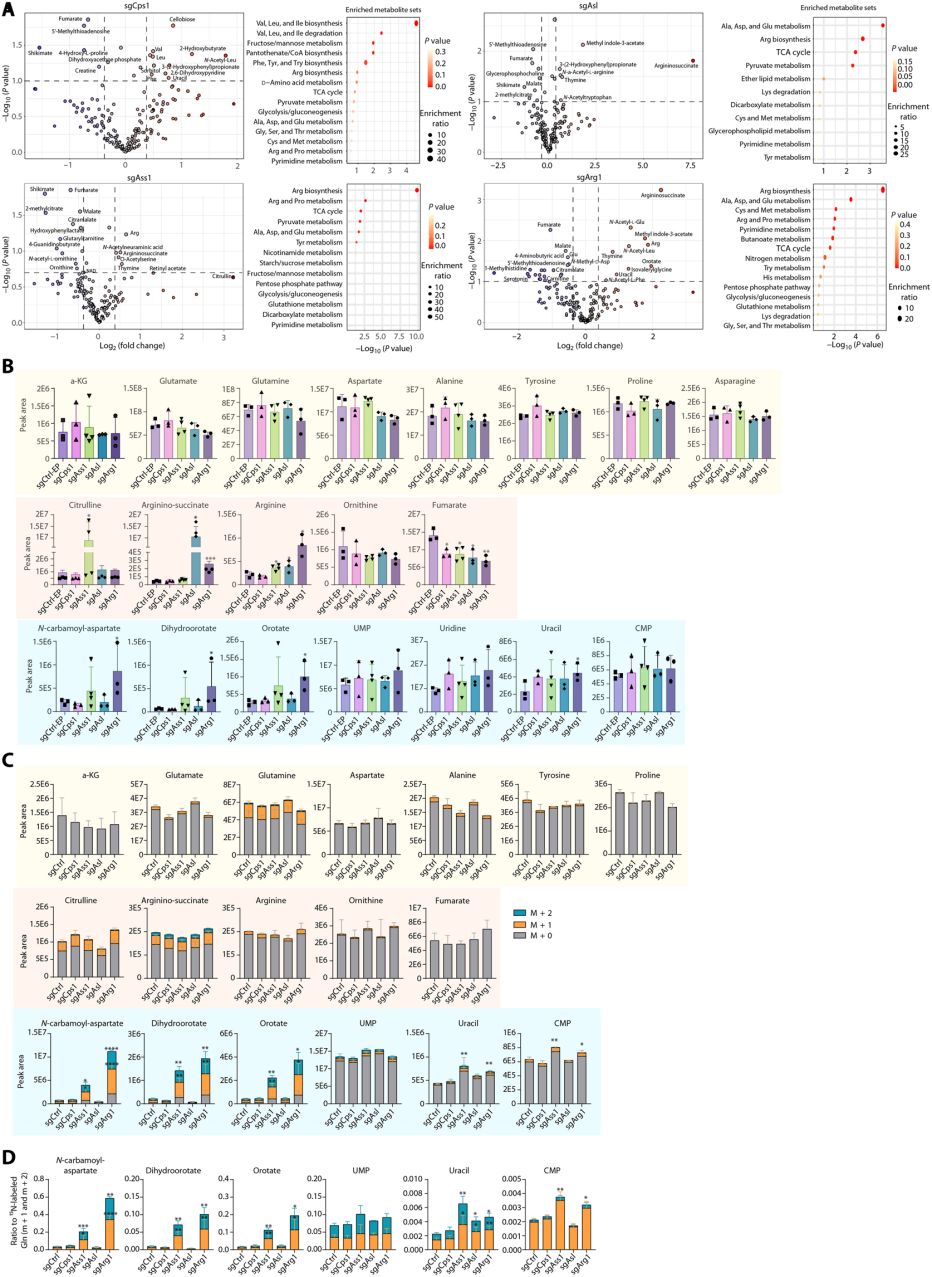
Silencing UCEs leads to abnormal amino acid metabolism and pyrimidine synthesis. (**A**) Untargeted metabolomics were performed using livers harvested from the sgUCE mice and their respective sgControl end-point (EP) mice. Volcano plots showing the metabolomic changes in sgUCEs liver compared with sgCtrl-EP liver and pathway enrichment analysis of significantly changed metabolites. (**B**) Abundance of indicated metabolites in sgCtrl–end-point (EP) livers and sgUCE livers (*n* = 3 in each group). (**C**) ^15^N-ammonium chloride was administered via intraperitoneal bolus 2 weeks after SB-HTVI. Livers were harvested after 30 min and LC-MS performed. The abundance of ^15^N-labeled and total metabolites in sgCtrl and sgUCE livers are shown (*n* = 3 to 4 in each group). (**D**) Relative abundance of ^15^N-labeled metabolites normalized to ^15^N-labeled glutamine in sgCtrl and sgUCE livers (*n* = 3 to 4 in each group). Data are shown as the mean value ± SD in (B), (C), and (D). *P < 0.05; **P < 0.01; ***P < 0.001; ****P < 0.0001; ns, not significant. Statistical significance was determined by unpaired Student’s *t* test.

Like in the c-MET/β-catenin model, silencing individual UCEs in the c-MET/sgAxin1 model also showed increased ammonia burden (Fig. 4, C and G). We then performed untargeted metabolomics and ^15^N-NH_4_Cl tracing to determine the effect of UCE silencing on metabolism. We used end-point mice to perform untargeted metabolomics. LC-MS showed that comparing with their respective sgControl mice, sgUCEs all shared altered arginine biosynthesis, NEAA metabolism, tricarboxylic acid (TCA) cycle, glucose metabolism, and pyrimidine metabolism (Fig. 5A). Some noticeable metabolites include elevated uracil in sgCps1, elevated argininosuccinate and arginine in sgAss1, elevated arginine in sgAsl, and elevated orotate and uracil in sgArg1 mice (Fig. 5A). Looking more closely at the three major ammonia metabolic fates, no significant changes in the glutamate/glutamine pathway were observed (Fig. 5B). In the UC pathway, sgAss1 led to accumulation of Ass1’s immediate substrate citrulline; sgAsl and sgArg1 led to accumulation of their substrates argininosuccinate; sgArg1 had a most significant accumulation of arginine, and the UC products ornithine and fumarate were generally lower in all four sgUCE groups (Fig. 5B). These data are consistent with the functions of each of the UCEs. When looking at the pyrimidine synthesis pathway, sgAss1 and sgArg1 mice showed marked increase of pyrimidine metabolites *N*-carbamoyl-aspartate, dihydroorotate, and orotate, while all four sgUCE groups showed a trend of increased uridine monophosphate, uridine, uracil, and CMP (Fig. 5B).

To further dissect the role of the altered nitrogen metabolism in tumorigenesis, we performed ^15^N-NH_4_Cl tracing in mice at early stage (2 weeks) of oncogene expression, similar to what we performed in the c-MET/β-catenin mice (Fig. 3). Like in the c-MET/β-catenin mice, no obvious difference was observed in ammonia assimilation in the glutamate/glutamine cycle upon the UCE silencing (Fig. 5C). Unlike in the untargeted metabolomics of the end-point mice (Fig. 5B), the UC metabolites did not show obvious difference in ^15^N-labeling (Fig. 5C), likely because only a small portion of hepatocytes had received c-MET/sgAxin1 oncogenes. Nonetheless, even at the early stage, ^15^N incorporation into the pyrimidine synthesis pathway was drastically higher in sgAss1 and sgArg1 mice (Fig. 5C), and when normalized to labeled glutamine, there was a trend of increased ^15^N incorporation into pyrimidine synthesis in sgAss1, sgAsl, and sgArg1 mice (Fig. 5D). sgCps1 did not show significant changes in the UC and pyrimidine metabolites because Cps1 catalyzes the production of carbamoyl phosphate that is the substrate for both UC and pyrimidine synthesis. It remains to be determined how sgCps1 rewires nitrogen metabolism to promote HCC development. Together, silencing individual UCEs in the c-MET/sgAxin1 model promotes HCC development, which is facilitated by increased pyrimidine synthesis and altered amino acid metabolism, particularly in the sgAss1, sgAsl, and sgArg1 mice, in a manner like that observed in the c-MET/β-catenin mice.

### LPD alleviates HCC development

**Fig. 6.**
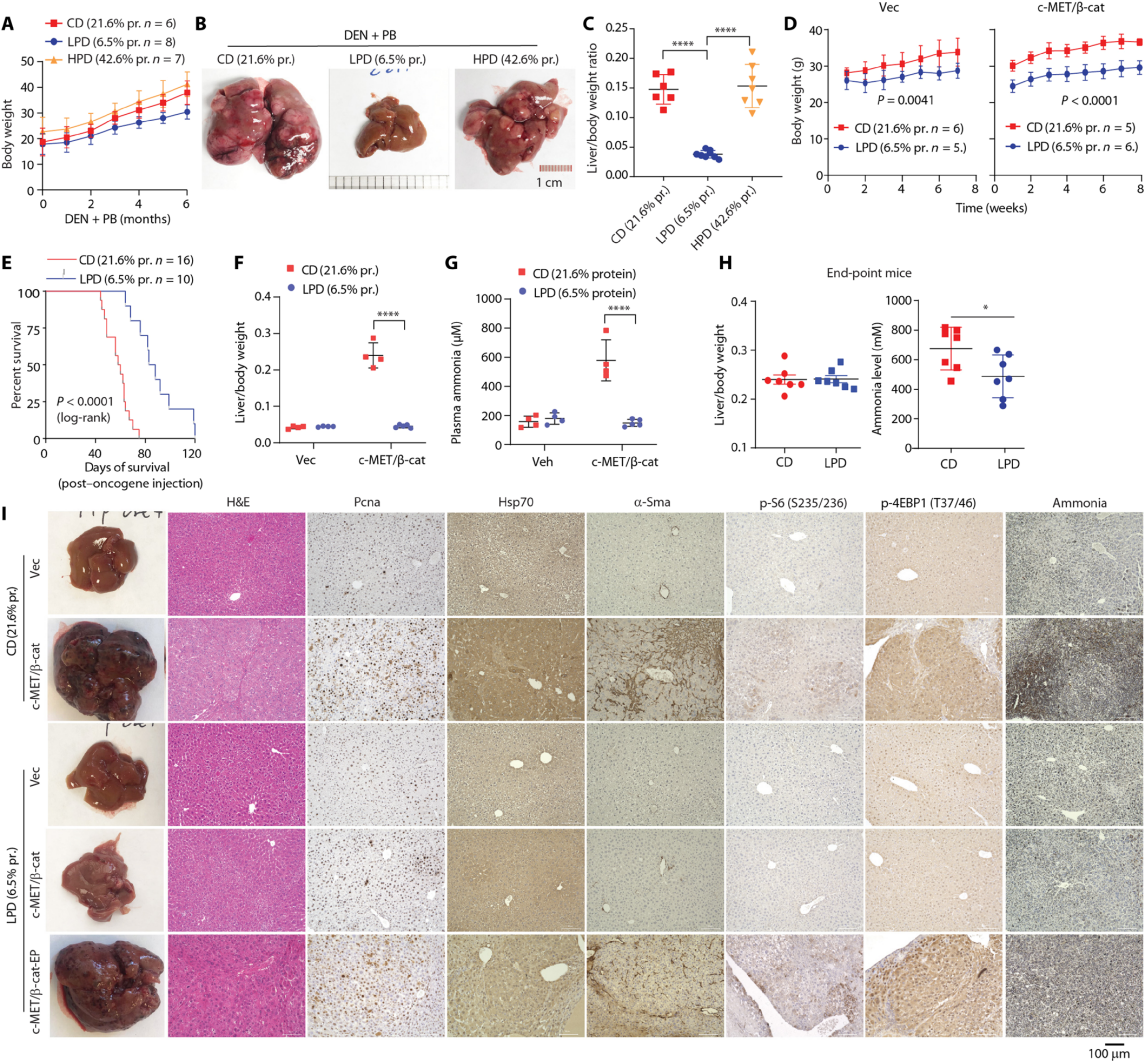
LPD delays HCC development. (**A** to **C**) Fourteen-day-old male mice were injected with DEN, then started being fed with CD, LPD, or HPD, with PB in drinking water. Body weight was measured every other week (A). Representative liver gross images (B) and liver/body weight ratio (C) are shown (*n* = 6 or 7 in each group). (**D** to **H**) Eight-week-old male mice were fed with CD or LPD for one week and then injected with vector control (Vec) or c-MET/ΔN90–β-catenin via SB-HTVI. Mice were kept on the same CD or LPD diet. Body weight (D) and Kaplan-Meier survival curves (E) are shown. When the CD-fed c-MET/ΔN90–β-catenin group reached the end point, they were harvested together with other groups. Liver/body weight ratio (F) and plasma ammonia level (G) were measured (*n* = 4 to 5 in each group). (H) CD- and LPD-fed mice, both harvested at their end points, (I) Representative gross images, H&E staining, and IHC for UCEs, Pcna, Hsp70, α-Sma, p-S6 (S235/236), p-4EBP (T37/46), and the Nessler’s staining for ammonia. Data are shown as the mean value ± SD in (A), (C), (D), (F), and (G). *P < 0.05; ****P < 0.0001; ns, not significant; Statistical significance was determined with unpaired Student’s *t* test in (C), log-rank test in (E), and two-way analysis of variance (ANOVA) with Sidak’s multiple comparisons test in (F) to (H).

Clinical data show a strong correlation between defective UCEs, hyperammonemia, and HCC (*19*, *23*, *40*, *41*). High dietary protein intake has been shown to increase blood ammonia level, which is exacerbated in patients with liver diseases including cancer (*5*, *42*, *43*). Restricting dietary protein intake has long been considered as a treatment for hepatic encephalopathy (*44*–*46*). Our above results point to a tumor-promoting role of defective ammonia clearance in HCC. It is then with great interest to determine whether lowering nitrogenous waste burden can help mitigate HCC. To this end, we went on to test the effect of lowering dietary protein on HCC development in two mouse models: the one induced by the carcinogen DEN and the other induced by c-MET/β-catenin, as they both showed decreased UCE expression (Fig. 1, D to F). In the DEN model, the chemical carcinogen DEN was used to treat 14-day old mice, which has been characterized to have histology and gene expression similar to human HCC (*47*–*49*). After weaning, mice were fed with chow diet (CD, Inotive TD 91352: % kcal from: 21.6 protein, 65.4 carbohydrates, and 13.0 fat), low-protein diet (LPD, Inotive TD 90016: % kcal from: 6.5 protein, 80.4 carbohydrates, and fat 13.1), or high-protein diet (HPD, Inotive TD 90018: % kcal from 42.6 protein, 44.3 carbohydrates, and 13.1 fat). While LPD modestly reduced weight gain, HPD led to slight increased weight gain comparing with the CD

(Fig. 6A). Although HPD did not significantly affect the tumor burden, strikingly, LPD markedly reduced the tumor growth (Fig. 6, B and C). We then compared the effect of LPD on HCC development induced by oncogenes c-MET/β-catenin. Similar to the DEN model, switching from CD to LPD led to reduced weight gain (Fig. 6D) and markedly prolonged survival and reduced tumor burden (Fig. 6, E and F), which was accompanied by decreased plasma ammonia level (Fig. 6G). LPD-fed mice harvested at their end points, despite similar tumor burdens as the CD-fed mice (Fig. 6H), had lower plasma ammonia level (Fig. 6H). Histology examination and IHC showed that mice fed with LPD had decreased hepatocyte proliferation (PCNA), liver fibrosis (α-SMA), mTOR signaling (phospho-S6 and phospho-4EBP1) (Fig. 6I). Consistent with the plasma ammonia level (Fig. 6H), end-point LPD liver showed decreased Nessler’s staining (Fig. 6I). Together, these results indicate that LPD can decrease HCC growth, likely in those with defective UCE expressions.

**Fig. 7.**
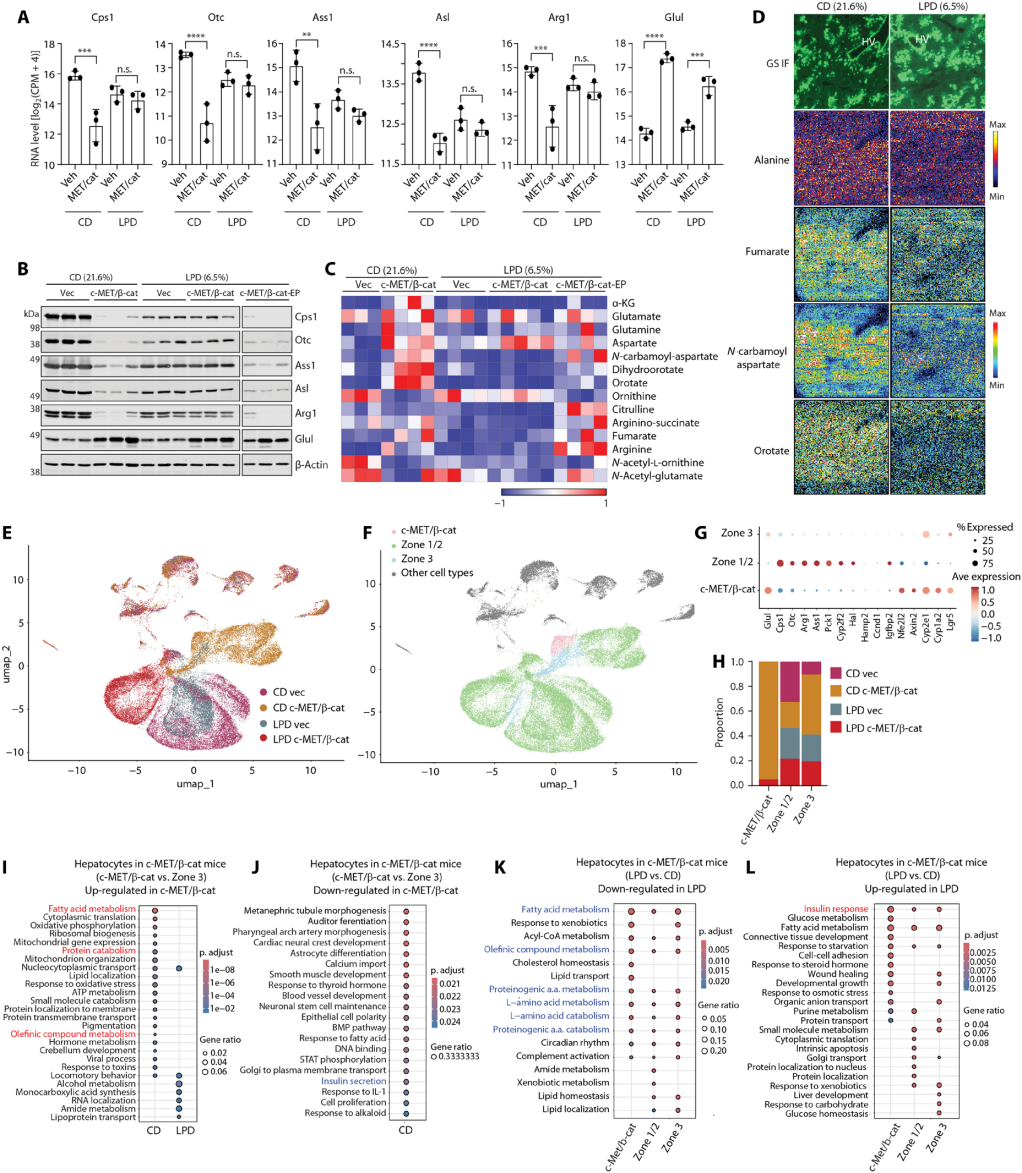
Low-protein diet alleviates nitrogen burden and altered nitrogen metabolism induced by c-MET/β-catenin. Vector control and c-MET/ΔN90–β-catenin mice fed with CD or LPD (*n* = 3 in each group) were harvested at the end point of the CD-fed c-MET/ΔN90–β-catenin mice. (**A**) RT-qPCR and (**B**) Western blot of indicated genes in liver from vehicle and c-MET/ΔN90–β-catenin injected mouse with CD or LPD (*n* = 3 in each group). **P < 0.01; ***P < 0.001; ****P < 0.0001. (**C**) Untargeted metabolomics was performed in indicated groups. Heatmap shows the relative abundance of indicated metabolites in livers from vehicle and c-MET/ΔN90–β-catenin–injected mice fed with CD or LPD (*n* = 4 in each group). (**D**) DESI-MSI data were acquired from the liver sections of c-MET/β-catenin–injected mice fed with CD and LPD (for 2 weeks) in negative ionization mode, followed by IF of GS expression to indicate zone 3 (HV) hepatocytes and those express the oncogenes. Note the highly increased fumarate, *N*-carbamoyl aspartate, and orotate in the CD mice, whereas alanine without drastic difference. (**E** and **F**) Pan-cell–type UMAP embeddings of cells (*n* = 42,277) colored by sample (E) and by zone [(F) with nonhepatocytes grouped together and colored gray] respectively. (**G**) Hepatocyte-specific dot plot of hepatocyte populations versus zonation markers. (**H**) Bar plot of relative sample proportions in each hepatocyte population. (**I** and **J**) Dot plot of GO enrichment terms versus diets in oncogene injected animals in the oncogene expressing versus zone 3 up-regulated (I) and down-regulated (J) directions, respectively. Only the CD is shown for the down-regulated direction as no significant terms were found for the low protein diet. (**K** and **L**) Dot plots of GO enrichment terms versus hepatocyte populations in oncogene-injected animals in the LPD versus CD down-regulated (K) and up-regulated (L) directions, respectively. For (I) to (L), pathways mentioned in the text are in bold with up-regulated pathways colored in red and down-regulated pathways colored in blue.

We performed bulk RNA-seq to compare liver tissues from four groups of mice: vector control fed with CD, c-MET/β-cat mice fed with CD, vector control fed with LPD, and c-MET/β-cat mice fed with LPD. Differential gene expression and gene set enrichment analyses were performed and identified two clusters that might help explain the differential tumor development in the CD and LPD groups (fig. S4). Cluster A (719 genes) were markedly up-regulated and cluster B (281 genes) down-regulated in the c-MET/β-cat mice fed with CD, yet the changes in expression were tempered in the oncogene LPD group. Cluster A was enriched in pathways including cell division, survival, DNA damage response, and immune response, consistent with the rapid tumor progression in the CD group and slow tumor progression in the LPD group (fig. S4). On the other hand, cluster B was enriched in pathways of carboxylic acid metabolism, triglyceride metabolism, and nitrogen metabolism (fig. S4). The down-regulation of nitrogen metabolism in the CD c-MET/β-cat group was consistent with our earlier results that expression of the UCEs were suppressed by β-catenin (Figs. 2 and 3), and the tempered changes of UCE expression in the LPD c-MET/β-cat group were confirmed by RT-qPCR and immunoblotting (Fig. 7, A and B). The LPD mice harvested at the end point also showed decreased UCE expression (Fig. 7B), indicating that decreased UCE expression induced by β-catenin is a hepatocyte-intrinsic effect.

Consistent with the decreased expression of nitrogen metabolism genes in the LPD mice, untargeted bulk metabolomics showed that the drastic changes of UC metabolites (citrulline, argininosuccinate, arginine, fumarate, and ornithine) and the pyrimidine metabolites (*N*-carbamoyl aspartate, dihydroorotate, and orotate) in the oncogene CD group were also tempered in the oncogene LPD group (Fig. 7C). Even in the end-point LPD mice, levels of the pyrimidine metabolites were virtually lower than in the end-point CD mice (Fig. 7C). The liver sections of early-stage c-MET/β-catenin mice fed with CD and LPD, which were harvest at 2 weeks of oncogene expression, were examined by desorption electrospray ionization MS imaging (DESI-MSI) for the spatial distribution of metabolites. While immunohistofluorescence (IF) of GS expression indicated the zone 3 (hepatic vein) cells and those expressed the oncogenes, DESI-MSI showed highly increased fumarate, *N*-carbamoyl aspartate, and orotate in the CD mice (Fig. 7D), similar to that detected by ^15^N-NH_4_Cl tracing (Fig. 3D). LPD led to drastic decrease of these UC and pyrimidine metabolites similar to that detected by bulk metabolomics (Fig. 7, C and D). Together, these data indicate that the nitrogenous waste burden can be reduced by dietary protein restriction and support the theory that attenuated nitrogen metabolic reprogramming can contribute to the development of HCC.

### snRNA-seq reveals reprogrammed hepatocytes in LPD-fed mice

To further interrogate the impact of LPD on the c-MET/β-catenin–expressing hepatocytes, we performed single-nucleus RNA-seq (snRNA-seq) analysis. Mice were fed on CD or LPD for 1 week, followed by SB-HTVI of c-MET/β-catenin. Two weeks post–SB-HTVI, 42,277 single nuclei were sequenced with 12,413 in the CD-vector control, 10,225 in CD c-MET/β-cat, 9707 in LPD-vector control, and 9932 in LPD c-MET/β-cat (Fig. 7E). Unbiased clustering resulted in 12 unique cell populations, among which around 77% of the cells were hepatocytes (fig. S5, A and B). While the two vector control groups (CD and LPD) showed overlapping hepatocyte distributions, the oncogene-injected mice had largely shifted hepatocyte distributions (Fig. 7E). Clusters of c-MET/β-catenin–expressing (pink), zone 3 (blue), and zone 1/2 (green) hepatocytes were identified (Fig. 7F), based on the expression pattern of human c-MET and β-catenin that were introduced by SB-HTVI (fig. S5C), as well as differential expression of the zonation-specific genes (Fig. 7G). As expected, oncogene-expressing hepatocytes were derived exclusively from c-MET/β-catenin–injected mice. Furthermore, the LPD c-MET/β-cat group had fewer oncogene-expressing hepatocytes than the CD c-MET/β-cat group (Fig. 7H), likely due to the slower proliferation of the oncogene-expressing hepatocytes in the LPD group. In contrast, zone 1/2 and zone 3 hepatocytes were evenly distributed across all four samples. The oncogene-expressing hepatocytes, which were mostly localized in zones 1 and 2 (Fig. 2C), were reprogrammed to have gene expression features like the zone 3 cells (high expression of Glul, Cyp2e1, Cyp1a2, and Lgr5, as well as low expression of the UCEs and Pck1, Cyp2f2, Hal, Igfbp2; Fig. 7G). This is consistent with a recent report of single-cell RNA-seq of the CTNNB1 S45Y mutant mice (*50*).

We compared c-MET/β-cat–expressing hepatocytes versus zone 3 hepatocytes in c-MET/β-cat–injected mice. Oncogene-expressing hepatocytes showed higher fatty acid metabolism, protein catabolism, and olefinic compound metabolism in the CD but not LPD mice (Fig. 7I), whereas several morphogenesis development and insulin secretion pathways were down-regulated in oncogene-expressing hepatocytes compared to zone 3 hepatocytes in the CD fed mice (Fig. 7J). When comparing CD versus LPD in the three groups of hepatocytes (c-MET/β-cat–expressing, zone 1/2, zone 3) from the c-MET/β-cat mice, all three cell populations showed down-regulation of fatty-acid metabolism, olefinic compound metabolism, and several amino acid metabolism pathways in the LPD mice compared with the CD mice (Fig. 7K), opposite of CD-associated up-regulation of these pathways in oncogene-expressing hepatocytes compared to zone 3 hepatocytes. In contrast, cholesterol homeostasis and lipid transport were specifically enriched in oncogene-expressing hepatocytes, while lipid homeostasis and localization were enriched in zone 1/2 and zone 3 hepatocytes. On the other hand, higher expression of insulin response genes was observed in the LPD mice across all hepatocyte populations, with glucose metabolism and steroid hormone response pathways uniquely enriched in oncogene-expressing hepatocytes (Fig. 7L). Together, these results indicate that while oncogenic β-catenin reprograms zone 1/2 hepatocytes into zone 3-like hepatocytes and increases fatty acid metabolism, protein catabolism, and olefinic compound metabolism and decreases insulin-related gene expression, dietary protein restriction is associated with transcriptional changes in the opposite direction for the same pathways across all hepatocytes. Concurrently LPD also differentially alters lipid and glucose metabolism pathways in oncogene-expressing versus normal hepatocytes. These effects may underlie the protective effect of LPD in HCC development.

## DISCUSSION

In this study, we show that, mirroring the widespread yet heterogenous down-regulation of the UCEs in patients with HCC, a collection of HCC mouse models displayed divergent UCE expression levels, with substantial repression in several carcinogenic and oncogenic models including those driven by β-catenin but not by silencing the tumor suppressor Axin1 (Fig. 1). Along with the early onset of UCE down-regulation, the β-catenin HCC model displayed a progressive elevation of blood and hepatic ammonia levels. Untargeted metabolomics, ^15^N-ammonium chloride tracing, and transcriptomics characterization showed that reprogramming of amino acid metabolism increased nitrogen incorporation into pyrimidine synthesis in the β-catenin mice (Figs. 2 and 3). Silencing each of the UCEs Cps1, Ass1, Asl, or Arg1 (silencing of Otc unsuccessful) in the c-MET/sgAxin1 model, where the UCEs were only modestly affected (Fig. 1, D to F), resulted in elevated ammonia burden, together with reprogramming of amino acid metabolism and pyrimidine synthesis, supporting a causative role of defective UC in promoting HCC (Figs. 4 and 5). Dietary protein restriction in both the DEN/PB and the c-MET/β-catenin models greatly reduced the ammonia burden and slowed tumor progression (Figs. 6 and 7). Therefore, our study indicates that impaired nitrogenous waste handling is a risk factor of HCC. As defective UC can be an inherited genetic lesion or commonly caused by liver steatosis and oncogenic mutations, it may be imperative to lower dietary protein intake in the patients with high-risk of UC deficiency and oncogenic transformation.

Our study has systematically evaluated the UCE loss of function using in vivo spontaneous HCC mouse models. Several studies have explored the roles of UC in HCC (*22*–*31*). However, the results have been context dependent and controversial, largely because the UCEs are often already down-regulated in most HCC mouse models; therefore, it is difficult to study the loss of function of the UCEs. Here, we find that the c-MET/sgAxin1 model retains decent levels of the UCEs, which enabled us to silence each of the UCEs to study their impact on HCC development. Although Axin1 is known to suppress tumorigenesis by promoting β-catenin proteasomal degradation, several studies have shown that the tumor suppressor function of Axin1 can be independent of β-catenin and that silencing of Axin1 does not lead to the induction of the well-known β-catenin transcription target GS (*33*, *34*). Our observation that sgAxin1 does not suppress the expression of the UCEs while β-catenin does is in line with the β-catenin–independent function of Axin1 silencing, whose precise underlying mechanism needs further investigation. Moreover, while many oncogenic events correlate with down-regulation of the UCEs in patients with HCC and mouse models, several conditions such as loss of tumor suppressors Trp^53^ and CDKN2A may lead to increased UCE expression (Fig. 1G) (*16*). It will be interesting to assess whether silencing the UCEs in these models can accelerate HCC development as in the sgAxin1 model.

While liver is the organ where the intact UC exists and functions, many nonhepatic tissues have partial UC activities that can be further dysregulated and promote oncogenesis via various mechanisms by altering the biosynthesis of pyrimidines, polyamines, nitric oxide, and proteinogenic and nonproteinogenic amino acids; by activating the mTORC1 growth signaling pathway; by supplying other metabolic pathways such as the TCA cycle and fatty acid synthesis; and by altering epigenetics, pH level, and the tumor microenvironment (*15*, *51*). Quite strikingly, untargeted metabolomics, ^15^N-ammonium chloride tracing, and transcriptomic analyses indicate that suppression of the UC in its entirety in the c-MET/β-catenin model led to the reprogramming of amino acid metabolism and pyrimidine synthesis. The increased pyrimidine synthesis upon oncogenic β-catenin expression is consistent with a previous report with ^15^N-gluamine tracing (*52*). These metabolic changes were also observed in the sgRNA silencing of individual UCEs in the c-MET/sgAxin1 model. Most significantly is the elevation of arginine observed in late-stage tumorigenesis in both the c-MET/β-catenin mice (Fig. 2F) and the c-MET/sgAxin1/sgUCE mice (Fig. 5, A and B). This observation is consistent with previous reports that arginine availability plays a critical role in HCC (*22*, *32*). The ^15^N-incorporation into arginine was not significantly different at the early stages of tumorigenesis (Fig. 3B and 5C), suggesting that the increased arginine level at the late stage is not from de novo arginine synthesis; rather, it may be from increased arginine uptake due to arginine auxotroph as a result of the UC block (*22*). The ^15^N-ammonium chloride tracing found a strong incorporation of the ^15^N-ammonic nitrogen into the pyrimidine precursor molecules *N*-carbamoyl aspartate, dihydroorotate, and orotate at the early stage of oncogenesis in the c-MET/β-catenin model and the c-MET/sgAxin1 with sgAss1 and sgArg1, which was observed at 2 to 3 weeks upon oncogene expression (Fig. 3 and 5C). The pyrimidine synthesis remained consistently high from early stage (2 to 3 weeks) up to end-stage tumors (Fig. 2F and 5B), in line with the theory that the pyrimidine synthesis pathway plays a causative role in promoting oncogenesis.

Unlike sgAss1 and sgArg1, silencing Cps1 and Asl in the c-MET/sgAxin1 mice led to little or minimal labeling of the pyrimidine metabolites despite their protumorigenic effects. It is expected that sgCps1 dampens the pyrimidine synthesis pathway because Cps1 catalyzes the rate-limiting step that condenses ammonia, bicarbonate, and ATP into CP, which is the substrate for the downstream UC events and pyrimidine synthesis. The failure of ^15^N incorporation into pyrimidine precursors in the sgCps1 liver is consistent with the protumorigenic role of CPS1 in lung cancer by maintaining the pyrimidine pool (*11*), yet it contradicts the protumorigenic function of CPS1-deficiency in HCC. Increased fatty acid oxidation (*23*) and decreased Asp level hence elevated DAG-PKC pathway (*24*) have been described in CPS1-deficient cells, which await to be further tested in the spontaneous HCC mouse models. On the other hand, ASL has been mostly described as protumorigenic in several types of cancer cells (*27*–*30*). Here, we show that Asl silencing led to a drastic elevation of its substrate arginine-succinate, as expected, and accelerated HCC growth (Fig. 5, A and B). However, unlike sgAss1 and sgArg1, sgAsl did not cause a drastic increase of ^15^N-incorporation into the pyrimidine precursors, asking for further investigation of the mechanisms underlying the protumorigenic role of Asl silencing.

On the basis of our findings that the failure of effectively removing nitrogenous waste exacerbates HCC development, we tested whether lowering dietary protein content can help reduce the nitrogenous waste burden and HCC development. The results show that feeding the HCC mouse models (c-MET/β-catenin and DEN/PB) with LPD (~6% calories from proteins) significantly slowed down HCC development and prolonged survival (Fig. 6). This beneficial effect of LPD is accompanied by a lower plasma and liver ammonia level (Fig. 6, G and H), less repressed UCEs and nitrogen metabolism (Fig. 7, A to C), and less changes in metabolites including several amino acids, UC metabolites, and pyrimidine synthesis (Fig. 7D). The drastic changes of carboxylic acid and triglyceride metabolisms in the oncogene CD group are reminiscent of recent reports that the carbon backbone of dietary protein contributes to hepatic de novo lipogenesis more than fat or carbohydrate and may promote insulin-resistance and MASLD (*53*, *54*). The increased fatty acid metabolism and oxidative phosphorylation detected by snRNA-seq in the CD oncogene hepatocytes is also consistent with the recent reports that connect β-catenin mutated tumor with fatty acid oxidation (*50*, *55*).

### Limitations

The regulation of UCE expressions and activity is highly divergent among different tissues and pathological conditions (*15*, *51*). Transcription regulation of the UCEs in the liver has been studied at the basal state and under hormonal regulation in response to dietary protein intake and liver steatosis (*56*, *57*). In healthy liver, high level of β-catenin correlates with low UCE expression in zone 3. A recent study showed that β-catenin can down-regulate ARG1 expression by suppressing liver-enriched transcription factors C/EBPA and FOXA1 in liver cancer cell lines (*58*). Nonetheless, precise mechanisms of the regulation of UCE expression, especially under pathological conditions, remain largely elusive.

While dietary protein is generally thought to be important in maintaining nutritional balance, preventing sarcopenia and cachexia, and boosting immune strength, lowering dietary protein has been gradually recognized to be beneficial in weight management, reducing nitrogen load in kidney and liver diseases, preventing hepatic encephalopathy, and extending life span (*59*). Dietary protein can also affect gut microbiota composition and function (*60*). In cancers, dietary protein restriction and systemic ammonia load have been described to have various effects in the malignancies of colon, breast, and liver, by modulating the mTORC1 signaling and immune invasion (*7*, *61*–*63*). Ammonia has been shown to promote cell survival/proliferation and tumorigenesis by inducing autophagy (*64*, *65*), lipogenesis (*66*), assimilating into amino acids and nucleotides (*12*), altering cellular pH, and inhibiting antitumor T cell activation (*61*). Therefore, while our study supports a causal relationship between β-catenin, defective UCE expression, and HCC development in a manner that is intrinsic to the oncogene-expressing hepatocytes, the effect of dietary protein and ammonia burden on various cancer development can be complex and multifaceted.

Moreover, our data show that oncogenic c-MET/β-catenin signaling and UCE silencing increase plasma and hepatic ammonia levels, and our ^15^N-ammonia tracing, bulk LC-MS, and DESI-MSI analyses demonstrate corresponding alterations in UC intermediates, amino acid pathways, and pyrimidine biosynthesis. These findings establish that ammonia can serve as a substrate for enhanced amino acid and pyrimidine metabolism. While these results are consistent with a causal contribution of ammonia to tumor progression, additional studies would further strengthen the causal link. To this end, strategies such as ammonia-consuming probiotics, ammonia scavenger, and genetic approaches of modulating other ammonia metabolic pathways can be considered.

## MATERIALS AND METHODS

### Antibodies

The antibodies used are as follows: CPS1 (Santa Cruz Biotechnology, sc-376190, RRID:AB_10985993), OTC (ProteinTech, 26470-1-AP, RRID:AB_2880528), ASS1 (Cell Signaling Technology, 70720, RRID:AB_2799790), ASL (NOVUS, NBP1-87462, RRID:AB_11031222), ARG1 (Cell Signaling Technology, 93668; RRID:AB_2800207; ProteinTech, 16001-1-AP, RRID:AB_2289842), glyceraldehyde-3-phosphate dehydrogenase (ProteinTech, 10494-1-AP, RRID:AB_2263076), β-actin (Cell Signaling Technology, #4967, RRID:AB_330288), β-tubulin (Cell Signaling Technology, #2146, RRID:AB_2210545), β-catenin (Cell Signaling Technology, #9562, RRID:AB_331149), GLUL (BD Biosciences, 610517, RRID:AB_397879), HSP70 (Cell Signaling Technology, #4872, RRID:AB_2279841), PCNA (Cell Signaling Technology, 13110, RRID:AB_2636979), α-SMA (Abcam, ab124964, RRID:AB_11129103), p-S6 S235/236 (Cell Signaling Technology, 4858, RRID:AB_916156), and p-4EBP1 T37/46 (Cell Signaling Technology, 2855, RRID:AB_560835).

### Chemicals

DEN (Sigma-Aldrich, N-0756), ^15^N-NH_4_Cl (Cambridge Isotope Laboratories, NLM-467-PK), ^15^N-NH_4_OAc (Cambridge Isotope Laboratories, NLM-177-PK), LC-MS–grade methanol (Fisher Chemical, A456-4), ultrapure water (Fisher Chemical, W6-212), sodium formate (Waters, Milford, MA, USA, 700008892-2) and leucine enkephalin standard (Waters, Milford, MA, USA, 700008893-3), and formic acid (Fisher Chemical, A117-50).

### Constructs

Plasmids used in SB-HTVI system to induce HCC are as follows: pT3-EF1α-c-MET (human c-MET or hMet, Addgene, 86498), pT3-EF1α-Myc-ΔN90–β-Catenin (Addgene, #31785), pT3-EF1α-Flag-YAP S127A (Addgene, 86497), and pX330-U6-Chimeric_BB-CBh-hSpCas9 (pX330, Addgene, #42230). To delete Axin1 and UCEs in mouse liver, sgRNA against Axin1 (NM_001159598.1), Cps1 (ATGTGACTACGAAGCGACAG), Ass1 (CTATGATGTCATCGCCTACC), Asl (CATGGCATCAGAGGTGTGT), and Arg1 (TATGGTTCTGGACTTGGCG) was cloned into pX330. All plasmids were purified using Plasmid Maxi Kit (Qiagen, 12163).

### Cell lines

Hep3B [American Type Culture Collection (ATCC) HB-8064], Hep G2 (ATCC, HB-8065), SK-Hep-1 (ATCC, HTB-52), THLE-2 (ATCC, CRL-2706), PLC/PRF/5 (ATCC, CRL-8024), SNU-387 (ATCC, CRL-2237), SNU-423 (ATCC, CRL-2238), SNU-449 (ATCC, CRL-2234), and AML12 (ATCC, CRL-2254) cells were obtained from ATCC. Huh-7 cells were obtained from Creative Bioarray (CSC-C9441L). Huh-1 (RRID:CVCL_2956) cell line was a gift from S. Zheng (Rutgers Cancer Institute), and the MHCC97-L (CVCL_4973) was a gift from W.-X. Ding (University of Kansas Medical Center).

### RNA extraction, cDNA synthesis, and qPCR

RNA of tissue was isolated, and revers transcription was done using Superscript III First Strand Synthesis system (Invitrogen). qPCR was performed with Power SYBR Green PCR Master Mix (Thermo Fisher Scientific) on StepOnePlus (Applied Biosystems) using following primers: mCps1-F: CCTTTCTGTGAAGGCAAAGAC, mCps1-R: GCTTCCGGGTACCCTCCTAA; mOtc1-F: ACACTGTTTGCCTAGAAAGCC, mOtc1-R: CCATGACAGCCATGATTGTCC; mAss1-F: CCCCAGATTAAGGTCATCGCT, mAss1-R: CAGCCTCATAGCTGATGTGCA; mAsl-F: GGGTTGGACAAGGTTGCTGA, mAsl-R: TCCGTGACCACCTGGTCGTT; mArg-F: CGTAGACCCTGGGGAACACTATAT, mArg-R: CTGTAAGATAGGCCTCCCAGA.

### RNA-seq and data analysis

RNA-seq was performed at the University of Texas Health San Antonio Genome Sequencing Facility. RNA-seq library was prepared with 500 μg of total RNA following the Illumina TruSeq stranded mRNA sample preparation guide. After quantification, the library was subjected to cBot amplification and subsequent 50–base pair (bp) single-read sequencing on the Illumina HiSeq 3000 platform using 100PE sequencing module.

The RNA-seq reads were mapped to the mouse genome reference [*Mus musculus* genome (UCSC mm10)] using HISAT2, gene-level quantification was counted using FEATURE COUNTS, differentially expressed gene (DEG) analysis was conducted using DEseq, and heatmap was generated using iDEP online tool https://bioinformatics.sdstate.edu/idep/. Functional enrichment analysis was performed using the Metascape online tool https://metascape.org/gp/index.html#/main/step1.

### Single-nucleus RNA-seq

#### Nuclei isolation

Bl/6 mice were fed with CD or LPD for 1 week, injected with c-MET/β-catenin plasmids via SB-HTVI, and then kept on the same diets for two more weeks. Frozen liver tissue was isolated for snRNA sequencing using the Chromium Nuclei Isolation Kit (PN-1000494, 10x Genomics) according to the manufacturer’s protocol. Briefly, the frozen tissue samples were homogenized with a pestle in lysis buffer and passed through a column. Next, debris was removed via centrifugation in debris removal buffer. The isolated nuclei were then washed and resuspended and loaded directly into the 10x Chromium platform.

#### Cell counting and quality control

Suspension and fluorescent dye (AO/PI) were mixed at ratio of 1:1 and incubated for 30 s, and the suspension mixed were added to the slide and assessed cell viability using CountStar. Viability enrichment was performed using the Dead Cell Removal Kit (130-090-101, Miltenyi Biotec) as per the manufacturer’s protocol. The cells were resuscitated to a concentration of 700 to 1200 cells/μl (viability, ≥85%) in a final solution of 1× phosphate-buffered saline (PBS) + 0.04% bovine serum albumin before loading on the 10x Genomics Chromium platform.

#### Library construction and sequencing

A total of 10,000 cells were used to prepare snRNA-seq libraries. Chromium Single-cell 3’ Library and Gel Bead Kit V3.1 (10x Genomics, PN1000268) was used to generate single-cell gel beads in emulsion (GEMs). The captured cells were lysed, and the released RNA was reverse-transcribed with primers containing poly-T, barcode, unique molecular identifiers and read 1 primer sequence in GEMs. Barcoded cDNA was purified and amplified by PCR. The adapter ligation reaction was performed to add sample index and read 2 primer sequence. After quality control, the libraries were sequenced on Illumina Novaseq 6000 platform in 150-bp pair-ended manner (Berry Genomics Corporation, Beijing, China).

#### Preprocessing, quality control, and dimensionality reduction

We used CellRanger to align sequencing reads to a custom genome constructed by adding the human c-MET and ꞵ-catenin genes to the mm10 genome. We then analyzed the data using Seurat (v5) and prepared the data using the standard Seurat preprocessing workflow. We removed cells with fewer than 200 genes, greater than 7000 genes, or greater than 21% of reads mapping to mitochondrial genes. We then performed data normalization and scaling using Seurat’s built-in functions NormalizeData and ScaleData to adjust for sequencing depth and standardize genes. We also selected the top 5000 variable genes for downstream analyses using the FindVariableFeatures function. After running principal components analysis (PCA), we used Harmony to correct for batch effects in the PCA embedding and ensure cells clustered on the basis of biological similarity. Last, we performed nonlinear dimensionality reduction using Uniform Manifold Approximation and Projection (UMAP) to produce a 2D visualization of the cells.

#### Cell type clustering and annotation

After visualizing the variation of the harmony components using an elbow plot, we selected the top 23 components to generate a *k* nearest neighbor (kNN) graph using *k* = 20 and then used the Louvain algorithm to cluster cells. We tested different resolutions between 0.2 and 1.0 and continued with a resolution parameter of 0.4, which was the lowest resolution sufficient to identify biologically meaningful cell types. We then annotated the clusters using canonical markers to identify cell types. We visualized the expression patterns of these markers using Seurat’s DotPlot and FeaturePlot functions. To identify liver progenitor and mesothelial cells, we supported our annotations by using Seurat’s FindMarkers function to generate lists of genes overexpressed in the clusters and then using ClusterMole to suggest annotations based on those DEGs. During the cell type annotation process, we also identified clusters of cells that strongly expressed markers of multiple cell types, which we classified as doublets, and removed them.

#### Hepatocyte subtype annotation

Following basic cell type annotation, we subset out the hepatocyte cells for further subtyping. For this smaller dataset, we repeated data normalization and scaling, variable feature selection, PCA, batch effect correction, and UMAP visualization. After selecting the top 18 Harmony components based on an elbow plot of the variation, we then repeated clustering, constructing a new kNN graph and using the Louvain algorithm to identify clusters. We tested multiple resolutions but proceeded with a resolution of 1.0, which was sufficient to identify the different subtypes we expected, specifically the oncogene-expressing subcluster. After clustering, we used canonical markers, specifically Glul, to annotate clusters based on zonation, which was sufficient to differentiate between zone 1/2 (Glul^−^) hepatocytes from zone 3 and oncogene-expressing (Glul^+^) cells. We then used a combination of approaches to further annotate Glul^+^ hepatocytes. First, we used the human c-MET and ꞵ-catenin gene constructs, which are expressed exclusively by cells taking in these constructs in oncogene-injected animals. The transcripts were detected in only a small number of cells, but these cells congregated almost entirely within just one cluster, which we identified as the oncogene-expressing cluster. We annotated the other Glul^+^ cells as zone 3 hepatocytes. Second, we calculated the relative proportions of each sample within each of the hepatocyte clusters. The proportion results supported our annotation since the cluster identified as the oncogene-expressing hepatocytes contained almost exclusively cells from the two c-MET/ꞵ-cat samples, while the clusters identified as zone 3 hepatocytes contained cells from all four samples. After the clusters were identified, the annotations were transferred back to the original Seurat object.

#### Proportion analysis

We calculated the relative proportions of the cell types within each of the samples, as well as the relative proportions of the samples within each of the hepatocyte subtypes, and visualized those proportion results with bar plots.

#### DEG and pathway analysis

After cell type and subtype annotation, we performed the following comparisons: CD versus LPD in oncogene-injected animals in the three populations of hepatocytes, and oncogene-expressing hepatocytes versus zone 3 hepatocytes in oncogene-injected animals fed with CD or LPD. To compare cell populations of interest, we used FindMarkers to generate lists of DEGs and visualized them in volcano plots using EnhancedVolcano. We selected genes with a *P* value (adjusted for multiple testing using the Bonferroni method) of less than 0.05 and an absolute log fold change of at least 0.5. We then proceeded with pathway analysis. We first divided up selected genes based on the sign of their log fold change to separate the genes expressed in each direction of a given comparison. Next, we used ClusterProfiler to perform overrepresentation analysis on the DEGs against the Gene Ontology database. Last, we filtered out redundant terms with the simplify method (with the cutoff parameter set to 0.5), producing a list of mostly nonintersecting enriched gene functions for each comparison, which we visualized using dot plots.

### Immunoblotting

Tissue lysate was prepared using radioimmunoprecipitation assay buffer with 0.1% SDS and protease inhibitors cocktail (MCE Chemicals). Thirty micrograms of protein was separated in SDS–polyacrylamide gel electrophoresis and transferred to nitrocellulose blotting membrane. Primary antibodies were incubated overnight at 4°C, and Alexa Fluor–conjugated secondary antibody was incubated for 1 hour at room temperature. Blot was acquired using the Odyssey Imaging System (LI-COR Biosciences).

### Ammonia measurement

Ammonia level was measured using Ammonia Assay Kit (Abcam, #ab83360) following the manufacturer’s instructions. For TIF, it should be diluted 10 times in advance. Five microliters of diluted TIF or plasma was mixed with 45 μl of ammonia assay buffer, and the standard and background control were also set up according to the manufacturer instruction. Fifty microliters of the Master Reaction Mix and Master Background Reaction Mix was added to the standard, background control, and sample wells, respectively. The reaction was mixed well and incubated in the dark at room temperature for 60 min, and the adsorption was measured on microreader at optical density at 570 nm.

### Nessler’s staining for tissue ammonia

Nessler’s staining was performed as previously described (*67*). Briefly, paraffin-embedded tissue was sectioned into 4 μm and hydrated through washing with alcohol. The sample was incubated with Nessler’s reagent (VWR, #101203-286) for 5 min and then washed with tap water for 10 s. The sample was counterstained with hematoxylin, dehydrated, and mounted with Permount for imaging.

### IHC and IF analyses

IHC and IF were performed as previously described (*68*). For IHC, after hydration, antigen retrieval was performed in 10 mM citrate buffer (pH 6.0) by heating the section for 10 min and then allowing it to sit for 30 min. The endogenous peroxidase activity was quenched in 3% H_2_O_2_ in MeOH. The section was blocked for 2.5 hours with 10% goat serum followed by primary antibody incubation overnight at 4°C. After washing, the section was incubated with biotinylated secondary antibody for 1 hour, and then streptavidin-biotin complex (Vectastain Elite ABC kit, Vector Laboratories) was applied for 30 min. Peroxidase substrate solution 3,3′-diaminobenzidine (Cell Signaling Technology, #8059S) was applied for proper time until desired color reaction is observed. The reaction was terminated by rinsing gently with distilled water. The section was then counterstained with hematoxylin, dehydrated, and coverslipped. For IF, after antigen retrieval, the section was permeabilized with 0.4% Triton X-100 for 10 min and blocked with 10% goat serum for 2.5 hour, followed by primary antibody incubation overnight at 4°C. After washing, the section was incubated with fluorescently conjugated secondary antibody for 1 hour, costained with 4′,6-diamidino-2-phenylindole, and coverslipped. Micrographs were take using the Keyence BZ-X710 microscope. Blindly chosen fields were taken, and representative images were shown.

### Mouse experiments

Because HCC/liver cancer is preponderant in males, 6- to 8-week old C57/Bl3 male mice (unless otherwise stated) were used for in vivo tumor–related studies. To avoid the effects of circadian rhythm and feeding status, unless specified otherwise, all mouse experiments were performed at approximately 2 p.m. after 5 hours of fasting in advance. The mice were grouped randomly. All mouse experiments were performed in compliance with the Institutional Animal Care and Use Committee guidelines at Rutgers University, Rutgers University IACUC protocol 15-048.

#### Isolation of plasma

Blood was collected into heparinized tubes through cardiac puncture, mixed well, and sat on ice. Blood was centrifugated for 15 min at 2000*g* at 4°C, and the supernatant was transferred into an Eppendorf tube, snap-frozen in liquid nitrogen, and stored at −80°C.

#### Isolation of TIF

Liver tumor >100 mm^3^ were excised and rinsed in cold PBS and blotted dry with filter paper. The tumor was then placed on 20-μm Spectra/Mesh Woven Nylon filters (Spectrum Labs, Waltham, MA, #148134) loosely affixed to the top of the conical tube using laboratory tape. The lid of the conical tube is placed over the top of the conical tube and affixed with tape. The tumor tissue was centrifuged for 10 min at 106*g* at 4°C. TIF was transferred into an Eppendorf tube, snap-frozen in liquid nitrogen, and stored at −80°C.

### SB-HTVI–induced HCC mouse model

HTVI was performed as previously described (*34*). Briefly, for c-MET/ΔN90–β-catenin and YAP/ΔN90–β-catenin models, 20 μg of each oncogene plasmid and ^1^/_25_ of the total oncogene of pCMV-SB10 plasmids were diluted to 2 ml using Vetivex Lactated Ringer’s (NDC: 17033-91-50) and filtered with 0.22-μm filter (Millipore, GSWP04700). Plasmid solution was injected within 5 to 8 s via the tailvein into randomized mice. For c-MET/sgAxin1 UCEs KO model, 20 μg of pT3-EF1α-c-MET, 40 μg pX330-sgAxin1, along with 40 μg of pX330-sgCtl or pX330-sg UCEs, and ^1^/_25_ of the total oncogene of pCMV-SB10 plasmids were diluted into the 2 ml of Vetivex Lactated Ringer’s solution for each mouse. Tissues of the other SB-HTVI–induced HCC models were previously described: AKT/Ras (*69*), AKT/NCID (*70*), c-MYC (*71*), and YAP/TAZ (*72*).

### Carcinogen-induced mouse tumor models

For the DEN/PB-induced HCC model, 14-day-old male mice were injected with DEN (5 mg/kg; Sigma-Aldrich, N-0756) via intraperitoneal injection. Seven days later, the mice were fed 0.05% PB in drinking water (*48*, *49*). Livers from DEN/PB-treated mice were harvested after 8 months. For the DEN/HFD-induced HCC model, 14-day-old male mice were intraperitoneally injected with DEN (15 mg/kg) and then fed an HFD (42% kcal from fat; Envigo, TD. 88137) 7 days later. Livers from DEN/HFD-treated mice were collected after 6 months.

### Dietary protein experiment

CD (Inotive TD 91352: % kcal from 21.6 protein, 65.4 carbohydrates, and 13.0 fat), LPD (Inotive TD 90016: % kcal from 6.5 protein, 80.4 carbohydrates, and fat 13.1), or HPD (Inotive TD 90018: % kcal from 42.6 protein, 44.3 carbohydrates, and 13.1 fat) were purchased. Randomized mice were fed with the conditioned diets for 1 week before SB-HTVI and continued with the same diets till the end of the experiments.

### In vivo stable isotope labeling

For ^15^N-ammonium chloride tracing, the mice were injected with ^15^N-NH_4_Cl [5 mmol/kg (1.25 M, 100 μl/25 g body weight)] in Hanks’ balanced salt solution (Sigma-Aldrich, H6648) intraperitoneally. Liver was collected and snap-frozen 30 min after injection and then applied to LC-MS.

### Liquid chromatography–mass spectrometry

Liver tissues were harvested and snap frozen in liquid nitrogen. Thirty to fifty milligrams of tissue was placed in 2-ml Eppendorf tube, pulverized using the CryoMill (Retsch), and then thoroughly mixed with 40% methanol: 40% acetonitrile: 20% water: 0.5% formic acid for extraction. The extraction was proceeded on ice for 10 min and then centrifuged for 10 min at 15,000*g* at 4°C. The supernatant was collected, and (final volume × 8.8)/100 of 15% NH_4_HCO_3_ was added to neutralize the acetic acid.

LC-MS was performed on a hydrophilic interaction chromatography coupled with electrospray ionization to the Q Exactive PLUS hybrid quadrupole-orbitrap mass spectrometer (Thermo Fisher Scientific) with a 2.1 mm–by–250 mm column (2.5-μm particle size, Waters, Milford, MA). Five microliters of sample was loaded and separated at a flow rate of 300 μl/min using a gradient of solvent A [95%/5% H_2_O/acetonitrile with 20 mM ammonium acetate and 20 mM ammonium hydroxide (pH 9.4)], and solvent B [20%/80% H_2_O/acetonitrile with 20 mM ammonium acetate and 20 mM ammonium hydroxide (pH 9.4)]. The metabolite features were processed using MAVEN software, and each metabolite abundance was normalized to tissue weight across the sample population. Data were analyzed using MetaboAnalyst V6.0 (https://metaboanalyst.ca/) and differential metabolites (|log_2_ (fold change)| > |log_2_(1.3)|, P < 0.01) were used for pathway enrichment analysis. Heatmap was graphed using Morpheus (https://software.broadinstitute.org/morpheus/).

### Desorption electrospray ionization MS imaging

DESI-MSI data were acquired from 10-μm-thick mice liver sections in negative ionization mode using SYNAPT XS High-Definition Mass Spectrometer equipped with a DESI XS source (Waters, Milford, MA, USA). Before DESI-MSI data acquisition, the mass spectra were calibrated externally using a 0.5 mM sodium formate solution in 2-propanol:water (90:10, v/v). The spray solvent (98% methanol containing 0.1% formic acid and 100 pg/μl of leucine enkephalin) was delivered at a flow rate of 2 μl/min, using an ACQUITY UPLC (Waters, Milford, MA, USA). DESI sprayer position was adjusted following previously described DESI stage parameters to achieve high sensitivity (*73*). DESI-MSI data were acquired using following parameters; capillary voltage: 1.3 kV, cone voltage: 30 V, source temperature: 150°C, heated transfer line temperature: 250°C, N_2_ gas pressure: 0.1 MPa, and at a mass/charge ratio (*m/z*) range of 50 to 1200. The data were acquired in high resolution mode at a pixel size of 20 μm by 20 μm. The scan rate was 40 μm/s, mass resolution was 20,000, and mass window was set at 0.04 Da. Leucine enkephalin peak (*m/z* 554.2615) was used for lock mass correction to improve mass accuracy. MassLynx (version 4.2) software from Waters (Milford, MA, USA) was used for data acquisition. HDImaging (version 1.7) from Waters (Milford, MA, USA) and IMAGEREVEAL, Shimadzu (version: 1.31.0.12906) were used for data analysis.

### Statistical analysis

Data were analyzed using GraphPad Prism 8 software. Data were presented as means ± SD. Unpaired Student’s *t* test was used to compare between two groups, and two-way analysis of variance (ANOVA) with Sidak’s multiple comparisons test was used to compare the effects of two independent variables (factors) on a dependent variable among multiple groups. Log-rank (Mantel-Cox) test was used to analyze statistical significance in the Kaplan-Meier survival plots.
