# Supplementary material for: Impaired nitrogenous waste clearance promotes hepatocellular carcinoma
Source: Sci Adv. 2026 Jan 9;12(2):eaec0766. doi: 10.1126/sciadv.aec0766 (PMC12787525; doi:10.1126/sciadv.aec0766)
Supplement: Supplementary file 1 — Figs. S1 to S5 [file sciadv.aec0766_sm.pdf]

Science Advances
AAAS

Supplementary Materials for

Impaired nitrogenous waste clearance promotes hepatocellular carcinoma

Xinlu Han *et al.*

Corresponding author: Wei-Xing Zong, zongwx@pharmacy.rutgers.edu

Sci. Adv. **12**, eaec0766 (2026)
DOI: 10.1126/sciadv.aec0766

This PDF file includes:

Figs. S1 to S5

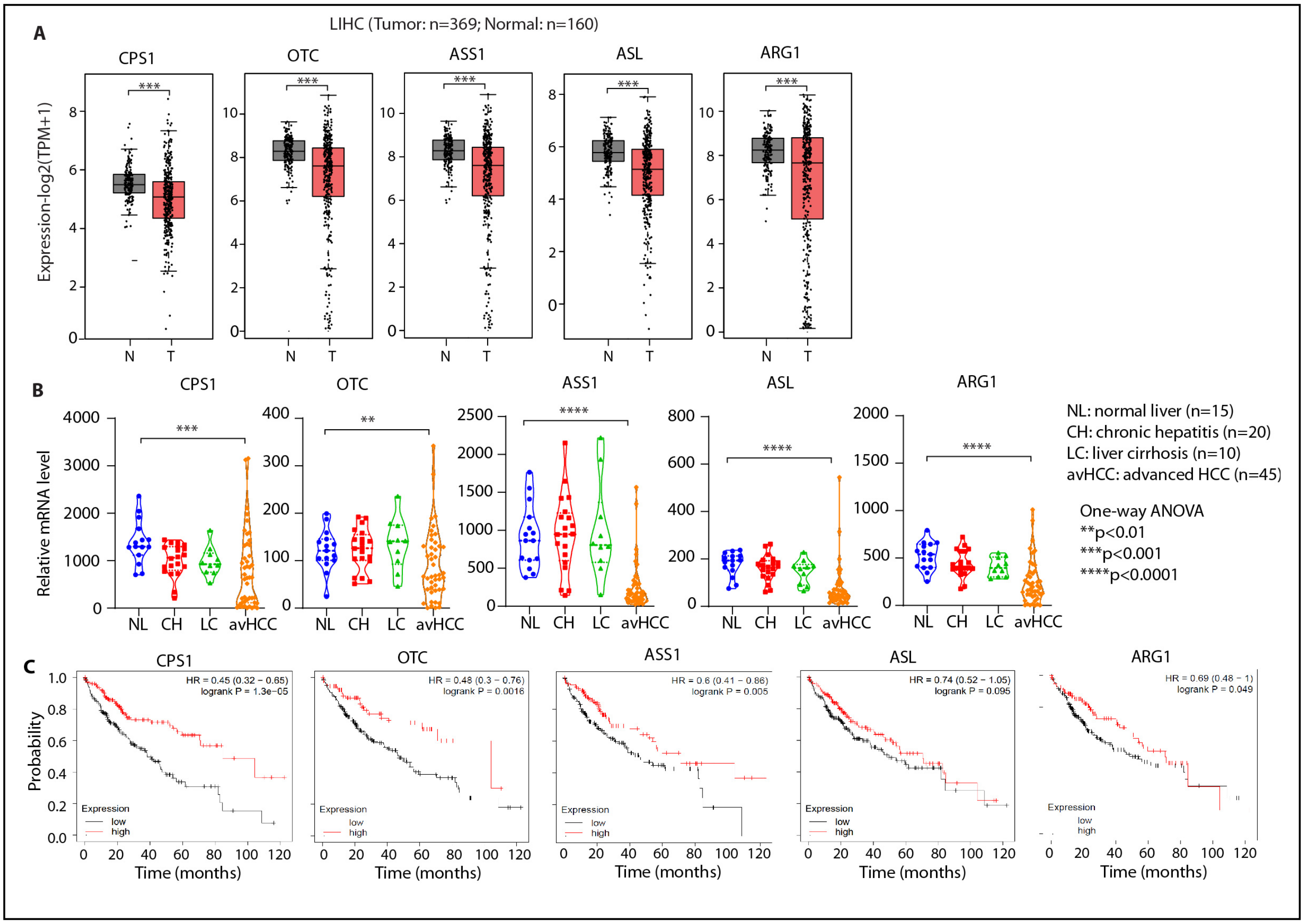

Fig. S1. Decreased expression of UCEs in liver cancer patients. (A) Boxplot showing mRNA level of UCEs in liver normal (N) and tumor (T) tissues, expression data retrieved from TCGA database. **(B)** Violin plot showing relative mRNA level of UCEs in normal liver, chronic hepatitis, liver cirrhosis and advanced HCC. **(C)** Kaplan-Meier survival curves showing the overall survival of patients with high or low level of UCEs, with cutoff at 50%. Data are shown as the mean value ± *SD* in **(A)**. **p<0.05*, ***p<0.01*, ****p<0.001*; *ns*, not significant; Unpaired Student's t test in **(A)**, Ordinary one-way ANOVA with Tukey's multiple comparisons tests in **(B)**, log-rank test in **(C)**.

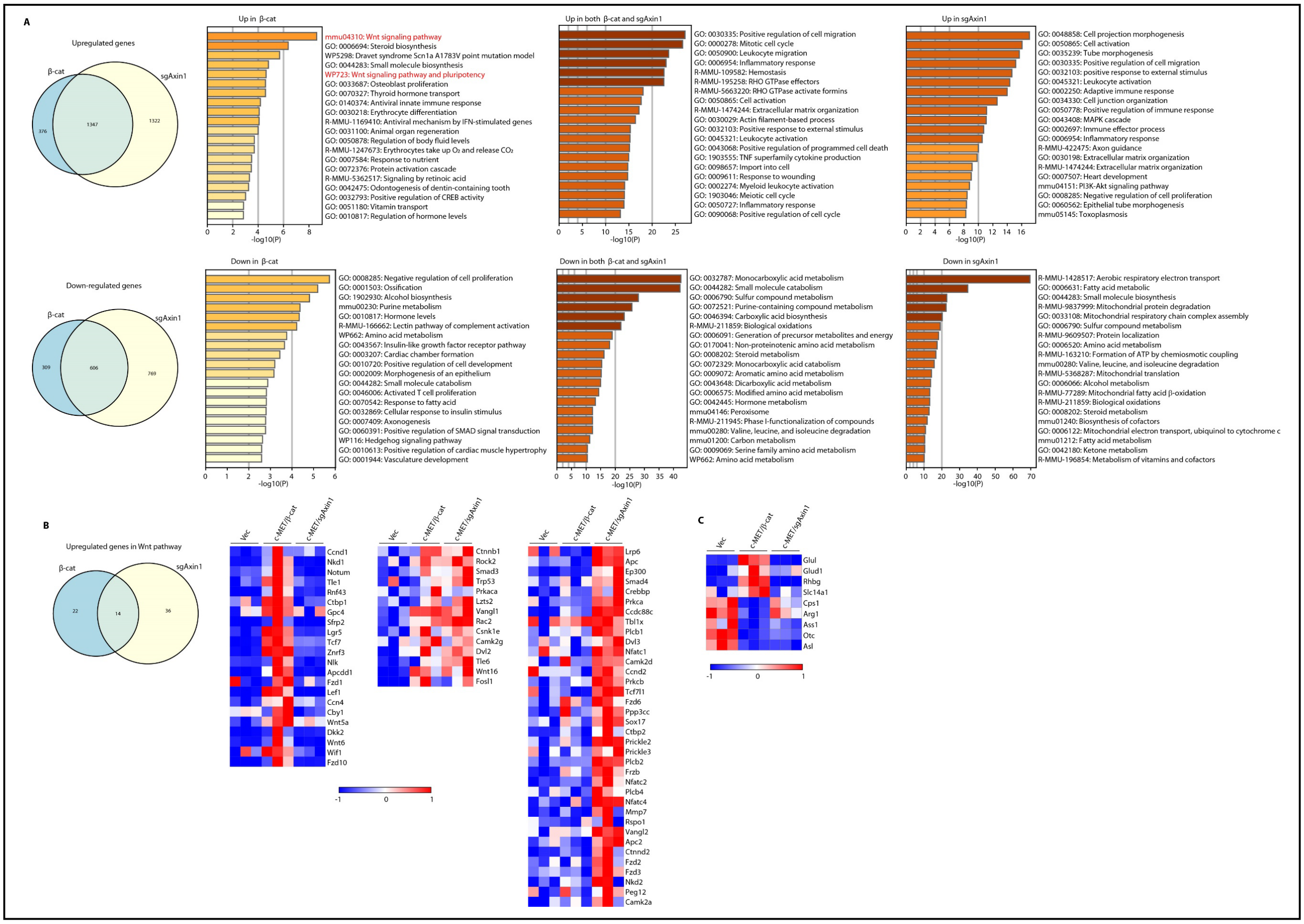

Fig. S2. β-catenin and sgAxin1 regulate differential gene transcription. c-MET/ΔN90-β-catenin or c-MET/sgAxin1 together with the Sleeping Beauty transposase plasmids were introduced into mice via HTVI-SB to induce HCC. Livers were harvested at the endpoint. Balk RNA-seq was performed. **(A)** (left) Veen diagram showing the overlap of upregulated genes and downregulated genes in c-MET/ΔN90-β-catenin tumor and c-MET/sgAxin1 tumor; (right) Gene enrichment analysis of genes upregulated or downregulated only in c-MET/ΔN90-β-catenin, in both c-MET/ΔN90-β-catenin and c-MET/sgAxin1, or only in c-MET/sgAxin1 livers. **(B)** (left) Venn diagram showing overlap of upregulated genes in the Wnt-signaling pathway in c-MET/ΔN90-β-catenin tumor and c-MET/sgAxin1 tumor. (right) Heatmap showing the relative level of upregulated Wnt genes in c-MET/ΔN90-β-catenin and c-MET/sgAxin1 tumor. **(C)** Heatmap showing the relative level of ammonia homeostasis genes in c-MET/ΔN90-β-catenin and c-MET/sgAxin1 livers.

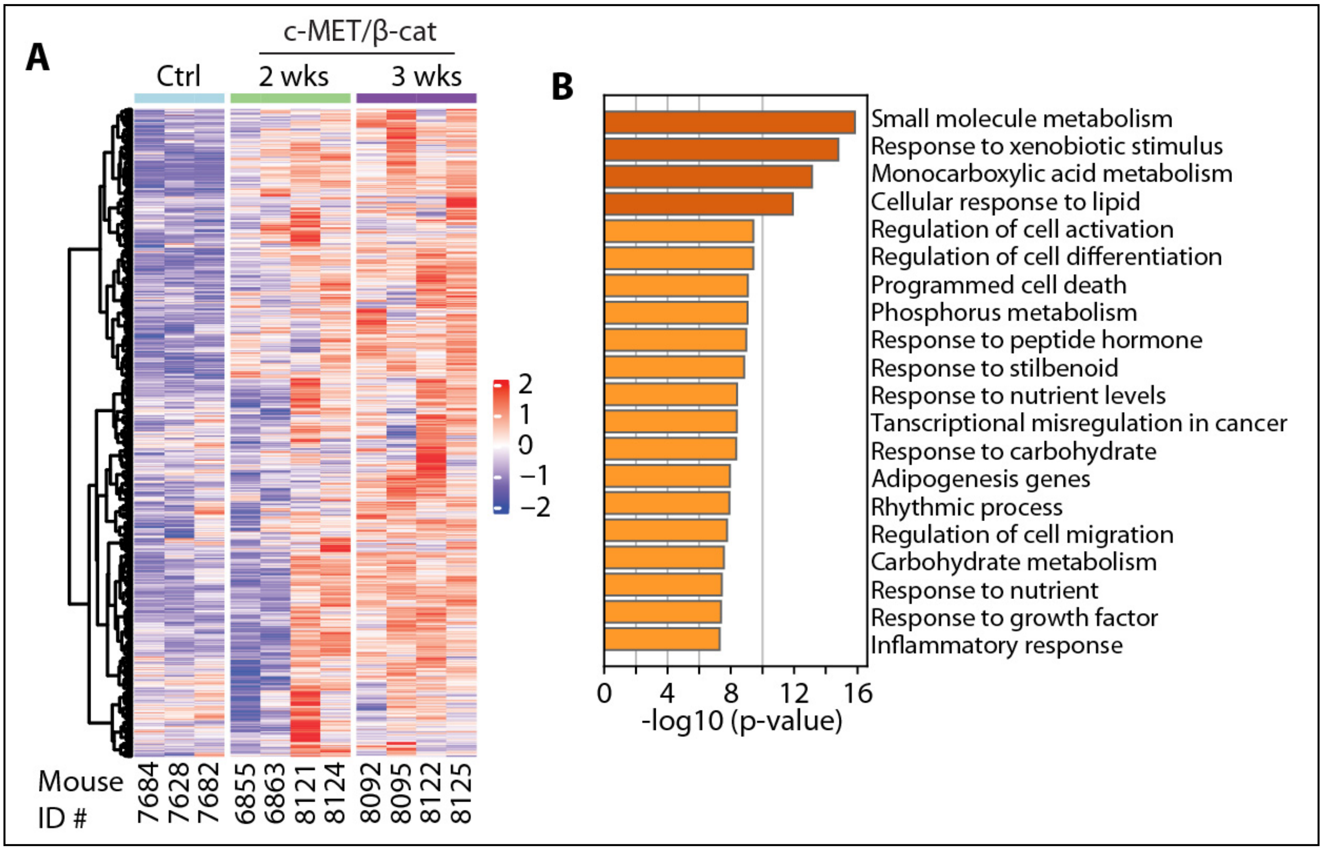

Fig. S3. Bulk liver tissue RNA-seq was performed using the vector control livers and that with c-MET/ΔN90-β-catenin SB-HTVI for 2 and 3 weeks. (A) Heatmap shows the upregulated genes in the oncogene-expressing livers. **(B)** Gene enrichment analysis of upregulated genes was performed by Metascape using GO biological process, KEGG pathways, Reactome gene sets library.

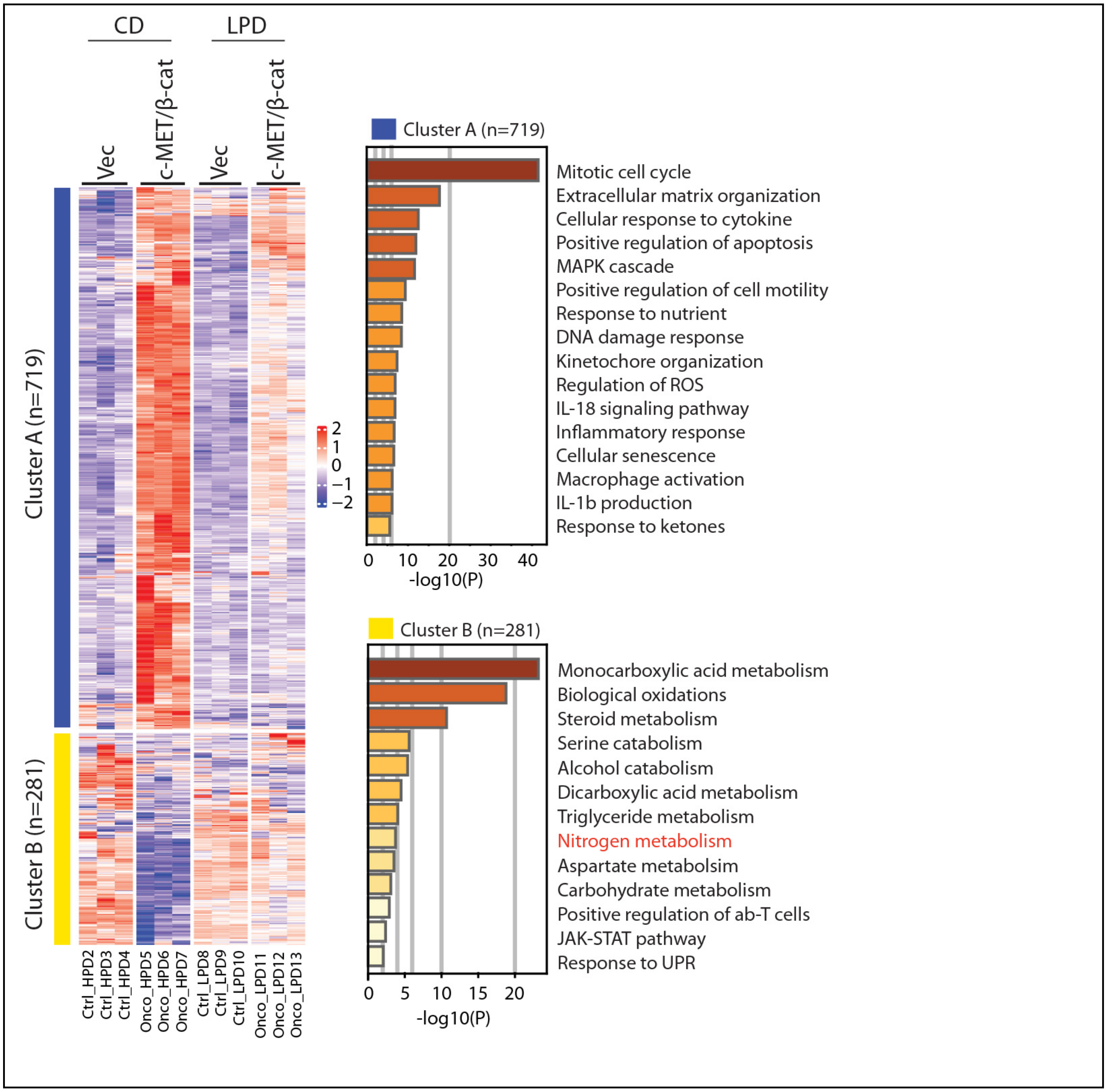

Fig. S4. Bulk RNA-seq of the CD and LPD-fed mice injected with vector control or c-MET/ΔN90-β-catenin by SB-HTVI. Vec and c-MET/ΔN90-β-catenin fed with CD or LPD (n=3 in each group) were harvested at the endpoint of the CD-fed c-MET/ΔN90-β-catenin mice. Liver tissue bulk RNA-seq was performed. The heatmap shows the genes that were markedly upregulated (Cluster A) and downregulated (Cluster B) in c-MET/ΔN90-β-catenin mice fed with CD, and reverted in the oncogene mice fed with LPD. (right) Gene enrichment analysis for genes in Cluster A and B.

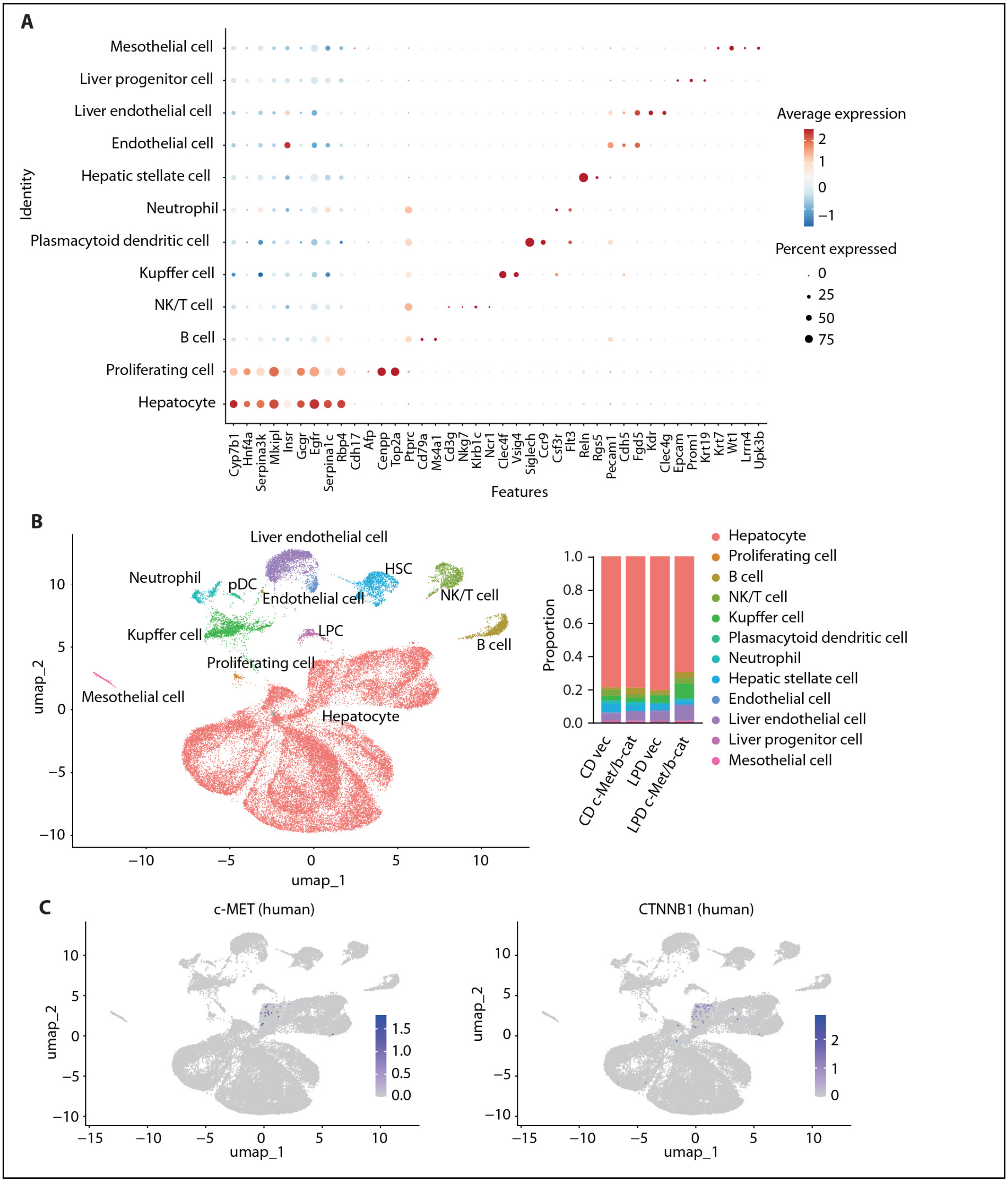

Fig. S5. Clustering of the snRNA-seq comparing the four groups of mice. (A) Dot plot of cell types vs canonical markers used to identify them. **(B)** Pan-cell-type UMAP embeddings of cells (n=42,277) colored by cell type, and bar plot of relative cell type proportions split by sample. **(C)** Pan-cell feature plots displaying expression of the human *c-MET* and *CTNNB1* genes.
